# EEG artifact removal using sub-space decomposition, nonlinear dynamics, stationary wavelet transform and machine learning algorithms

**DOI:** 10.3389/fphys.2022.910368

**Published:** 2022-08-24

**Authors:** Morteza Zangeneh Soroush, Parisa Tahvilian, Mohammad Hossein Nasirpour, Keivan Maghooli, Khosro Sadeghniiat-Haghighi, Sepide Vahid Harandi, Zeinab Abdollahi, Ali Ghazizadeh, Nader Jafarnia Dabanloo

**Affiliations:** ^1^ Occupational Sleep Research Center, Baharloo Hospital, Tehran University of Medical Sciences, Tehran, Iran; ^2^ School of Cognitive Sciences, Institute for Research in Fundamental Sciences, IPM, Tehran, Iran; ^3^ Bio-Intelligence Research Unit, Electrical Engineering Department, Sharif University of Technology, Tehran, Iran; ^4^ Department of Biomedical Engineering, Science and Research Branch, Islamic Azad University, Tehran, Iran; ^5^ Engineering Research Center in Medicine and Biology, Science and Research Branch, Islamic Azad University, Tehran, Iran; ^6^ Department of Clinical Neuroscience, Mahdiyeh Clinic, Tehran, Iran; ^7^ Department of Medical Genetics, Institute of Medical Biotechnology, National Institute of Genetic Engineering and Biotechnology (NIGEB), Tehran, Iran; ^8^ Sleep Breathing Disorders Research Center, Tehran University of Medical Sciences, Tehran, Iran; ^9^ Department of Psychology, Islamic Azad University, Najafabad Branch, Najafabad, Iran; ^10^ Department of Electrical and Computer Engineering, Islamic Azad University, Qazvin Branch, Qazvin, Iran

**Keywords:** EEG artifact removal, source separation, phase space reconstruction, noise reduction, subspace decomposition, stationary wavelet transform

## Abstract

Blind source separation (BSS) methods have received a great deal of attention in electroencephalogram (EEG) artifact elimination as they are routine and standard signal processing tools to remove artifacts and reserve desired neural information. On the other hand, a classifier should follow BSS methods to automatically identify artifactual sources and remove them in the following steps. In addition, removing all detected artifactual components leads to loss of information since some desired information related to neural activity leaks to these sources. So, an approach should be employed to detect and suppress the artifacts and reserve neural activity. This study introduces a novel method based on EEG and Poincare planes in the phase space to detect artifactual components estimated by second-order blind identification (SOBI). Artifacts are detected using a mixture of well-known conventional classifiers and were removed employing stationary wavelet transform (SWT) to reserve neural information. The proposed method is a combination of signal processing techniques and machine learning algorithms, including multi-layer perceptron (MLP), K-nearest neighbor (KNN), naïve Bayes, and support vector machine (SVM) which have significant results while applying our proposed method to different scenarios. Simulated, semi-simulated, and real EEG signals are employed to evaluate the proposed method, and several evaluation criteria are calculated. We achieved acceptable results, for example, 98% average accuracy and 97% average sensitivity in artifactual EEG component detection or about 2% as mean square error in EEG reconstruction after artifact removal. Results showed that the proposed method is effective and can be used in future studies as we have considered different real-world scenarios to evaluate it.

## 1 Introduction

EEGs containing brain electrical activity have become effective in different applications in all fields of science. These nonlinear and non-stationary signals can be employed to study the cognitive states or to diagnose mental disorders (Sanei and Chambers, 2007; Islam et al., 2016; Romo Vázquez et al., 2012; Klemm et al., 2009; Croft and Barry, 2000; Lagerlund et al., 1997; Li et al., 2006; Senthilkumar, 2008; Mumtaz et al., 2021; Yang et al., 2018; Cao et al., 2015; Rahman et al., 2015; Sai et al., 2018; Rodr´ıguez-Berm´udez and Garcia-Laencina, 2015; Mahajan and Morshed, 2015; Shoker et al., 2005; Jung et al., 2000; Vorobyov and Cichocki, 2002; Belouchrani et al., 1997; Delorme and Makeig, 2004; Min and Luo, 2009). Unfortunately, in most practical settings, EEGs are usually corrupted by environmental and physiological signals called EEG artifacts. Biological artifacts, including electromyogram (EMG), electrocardiogram (ECG), electrooculogram (EOG), eye blinking artifact, etc., levitate from non-cerebral sources in the human body. In contrast, environmental artifacts arise from external sources such as power line transmission, electric motors, electrode movement and etc., (Sai et al., 2018). Both types interfere with EEG signals easily and make interpretation and diagnosis difficult. Non-physiological artifacts are precluded by most EEG recording devices but biological artifacts like EMG and EOG still remain and need to be eliminated. This fact motivates us to propose a new method to reduce biological artifacts as interpreting corrupted EEGs is of great importance. Needless to say, artifact removal and noise suppression are inseparable parts in biological signal processing, and the more effective the methods are the more accurate the results will be. Therefore, there are several methods to deal with corrupted EEGs, such as linear filtering, autoregressive modeling, adaptive filters, blind source separation (BSS) based methods, wavelet transforms, principal component analysis (PCA) and etc., (Lagerlund et al., 1997; Croft and Barry, 2000; Li et al., 2006; Sanei and Chambers, 2007; Senthilkumar, 2008; Klemm et al., 2009; Romo Vázquez et al., 2012; Islam et al., 2016). Conventional methods like linear filters are not effective due to inherent overlap between artifacts and cerebral activity in the frequency domain (Sai et al., 2018; Rodr´ıguez-Berm´udez and Garcia-Laencina, 2015; Mahajan and Morshed, 2015; Shoker et al., 2005). BSS-based methods have been receiving a great deal of attention since they isolate artifacts into independent components (ICs) using subspace filtering (Sai et al., 2018). Second order blind identification (SOBI) algorithm, which is widely used in EEG preprocessing applications, utilizes the original EEG and time-shifted version(s) to exploit temporal information and estimate uncorrelated components (Cao et al., 2015; Rahman et al., 2015; Yang et al., 2018). BSS-based artifact removal consists of three major steps: 1) applying the source separation method, 2) source identification and artifact removal, and 3) channel reconstruction using a mixing matrix and remaining sources. Based on the previous experimental and analytical studies, these methods are useful tools in EEG artifact removal (Klemm et al., 2009; Romo Vázquez et al., 2012; Islam et al., 2016). Different articles have concluded that independent component analysis (ICA), introduced as a noise suppression tool for the first time in (Vorobyov and Cichocki, 2002), is one of the most robust methods in artifact elimination but is not very time fast. Among different BSS-based methods, second-order blind identification (SOBI) is reportedly one of the most effective methods and, at the same time, simple and practical. SOBI has been employed to remove artifacts in several studies. Several authors have found SOBI the most reliable and widely used approach (Lagerlund et al., 1997; Croft and Barry, 2000; Li et al., 2006; Ng and Raveendran, 2009; Klemm et al., 2009; Romo Vázquez et al., 2012; Sweeney et al., 2013; Islam et al., 2016). Several toolboxes like EEGLAB (Delorme and Makeig, 2004) have implemented SOBI due to its wide usage and efficiency. SOBI has been known as a superior method in comparison with ICA and most BSS methods. It should be noted that SOBI and other similar artifact removal methods in this family have their own shortcomings, which will be discussed later in this paper. Considering the advantages and disadvantages and also our application, we decided to use SOBI in this study to extract EEG sources. More detailed information about SOBI is brought in the following sections. To achieve reliable results, extracted sources should be identified to eliminate artifacts. Sources used to be visually identified by experts but this method often leads to insufficient EEG data for further analysis. Moreover, the origin of the artifacts is sometimes unknown. Thus source identification should be applied to achieve reliable neural sources. Manual identification methods are time-consuming and expensive. Researchers have proposed automated methods to identify extracted sources (Cao et al., 2015; Rahman et al., 2015; Yang et al., 2018). Mostly, sources are identified by classifiers using extracted features (Sai et al., 2018; Mumtaz et al., 2021). Since EEG is complex and chaotic, nonlinear analysis seems to be more successful in EEG artifact removal (Rodr´ıguez-Berm´udez and Garcia-Laencina, 2015). This motivates us to examine the phase space (of the extracted EEG sources) which is one of the most primitive EEG nonlinear analysis methods to identify extracted EEG sources and classify them into two groups containing neural sources and artifactual ones (Goshvarpour et al., 2016; Zangeneh Soroush et al., 2017; Zangeneh Soroush et al., 2018a; Zangeneh Soroush et al., 2018b; Zangeneh Soroush et al., 2018c; Zangeneh Soroush et al., 2018d; Zangeneh Soroush et al., 2019a; Zangeneh Soroush et al., 2019b; Zangeneh Soroush et al., 2020; Zangeneh Soroush, 2021). We introduce a new state space extracted from the EEG phase space. This new space is based on the angle values between points in the phase space and is called angle space (AS), resulting in a graphical illustration named angle plot (AP).

**FIGURE 1:**
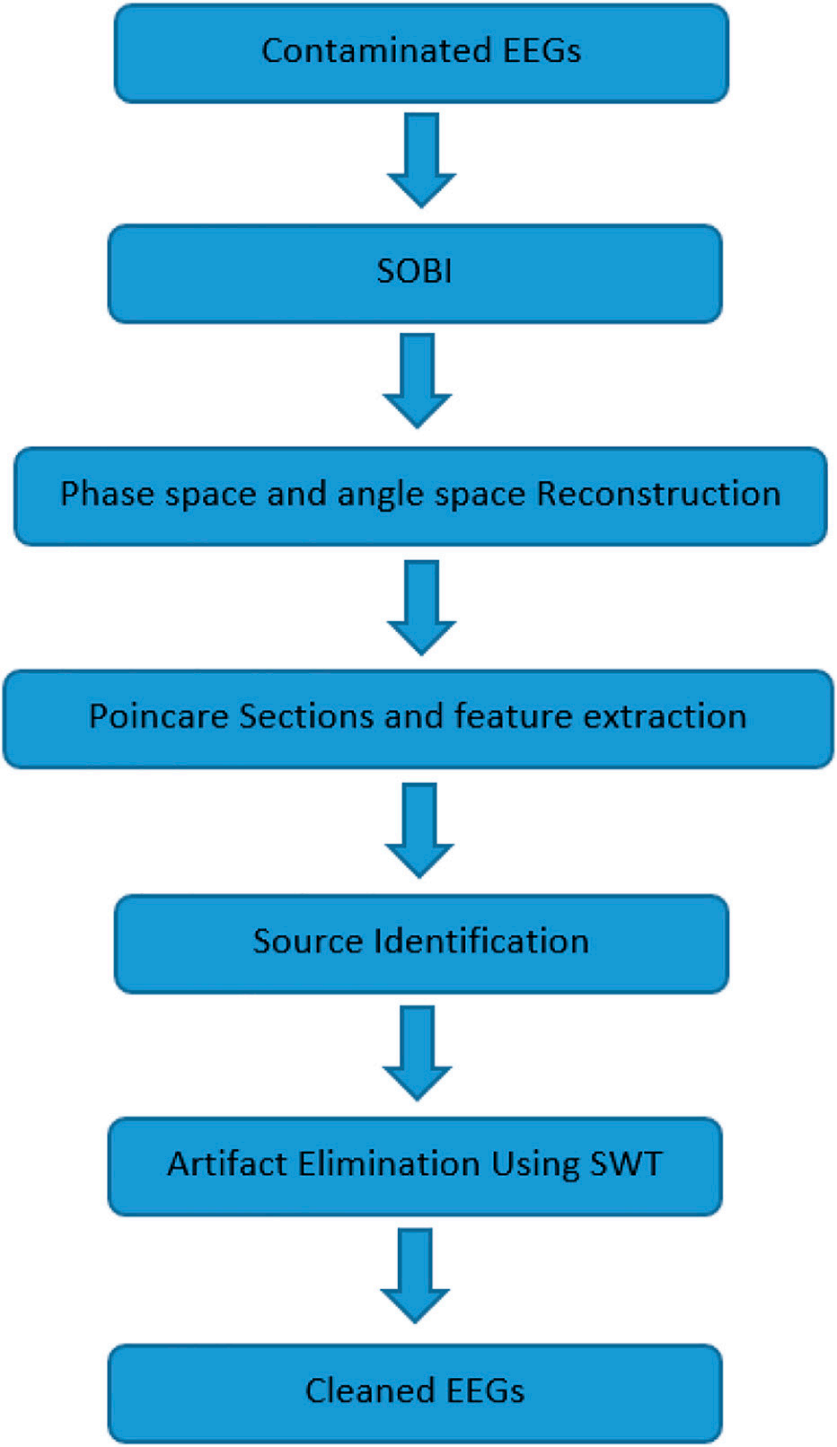
The block diagram of the proposed method.

Moreover, Poincare planes are effective to describe nonlinear signals (Belouchrani et al., 1997; Richman and Moorman, 2000; Seppänen et al., 2015; Sharma et al., 2015; Taskinen et al., 2016). So, Poincare planes are employed to quantify the APs. Extracted features from Poincare intersections are normalized, and then sources are classified using conventional classifiers such as multilayer perceptron (MLP) neural network, K nearest neighbor (KNN), Bayes and support vector machines (SVM). We also apply the ensemble of these classifiers to improve our classification results. Identified artifactual sources are fed into the artifact removal procedure using stationary wavelet transform (SWT). Several studies have claimed the advantages of SWT due to its ability to process non-stationary and nonlinear signals (Richman and Moorman, 2000). We employ SWT to prevent data loss since there is always information leakage to artifact components while using BSS methods. SWT can keep cerebral activity to a great extent compared to other wavelet transformations such as discrete wavelet transform (DWT) and continuous wavelet transform (CWT) (Romo Vázquez et al., 2012; Klemm et al., 2009; Croft and Barry, 2000; Lagerlund et al., 1997; Li et al., 2006; Senthilkumar, 2008; Mumtaz et al., 2021; Yang et al., 2018; Cao et al., 2015; Rahman et al., 2015; Sai et al., 2018; Rodr´ıguez-Berm´udez and Garcia-Laencina, 2015; Mahajan and Morshed, 2015; Shoker et al., 2005; Jung et al., 2000; Vorobyov and Cichocki, 2002; Belouchrani et al., 1997; Delorme and Makeig, 2004). Remained components are used to reconstruct the “clean” EEG. Not only is this method able to verify sources precisely, but it also can suppress artifacts effectively. Figure 1 shows the block diagram of the suggested method. Contaminated EEGs are separated into sources *via* the SOBI algorithm. Estimated sources are reconstructed in phase space. Reconstructed phase space is transferred into a new space called Angle Space (AS), and some quantifiers such as Poincare intersections are defined to describe phase space dynamics mathematically. Extracted features are fed into basic classifiers to identify sources. Real and simulated signals and artifacts are used in this study to assess the performance of the suggested method. Different criteria like classification performance (CP), relative root-mean-square error (RRMSE), Correlation Analysis (CA), and average mutual information (AMI) are defined to evaluate this method. Results show that the proposed method is successful.

This paper is organized as follows: “Section 2” represents material and methods. In “Section 3” you can find results. “Section 4” is dedicated to the discussion, and finally, the paper is concluded in “Section 5”.

## 2 Materials and methods

### 2.1 Blind source separation and second-order blind identification

BSS makes an effort to solve Eq. 1.

X(t)=AS(t) (1)

Where $X\left(t\right)=\left\{x_{1}\left(t\right).\;\ldots .\;x_{N}\left(t\right)\right\}$ and $S\left(t\right)=\left\{s_{1}\left(t\right).\ldots .\;s_{M}\left(t\right)\right\}$ represent observation signals for N channels (e.g., EEGs) and M estimated sources, respectively. A is called the mixing matrix and has the size of N∗M . In this model, EEGs are considered an instantaneous linear mixture of sources through an unknown mixing matrix of A (Wam et al., 2004).

SOBI algorithm is based on second-order statistics, and consists of two main stages 1) signals (i.e., EEGs): are zero-meaned, and whitening process is performed, and 2) a set of covariance matrices is constructed (Belouchrani et al. (1997) proposed SOBI for extracting correlated sources based on joint approximate diagonalization of a random set of time-lagged covariance matrices. The covariance matrix is defined based on Eq. 2.

$$R\left(q_{j}\right)=\frac{1}{C}\sum \bar{X}\left(t\right){\bar{X}}^{T}\left(t-q_{j}\right)$$ (2)

Where $\bar{X}\left(t\right)$ and ${\bar{X}}^{T}\left(t-p\right)$ are zero-meaned and time-delayed signals, respectively and q indicates time lags which are chosen as a set of different values instead of a single time lag to improve the time-efficiency of SOBI. C is the number of considered time lags. Sources are supposed to be mutually uncorrelated and stationary. Reportedly, SOBI is capable of functionally separating sources which are physiologically interpretable (Vigário and Oja, 2000; Tang et al., 2002a; Tang et al., 2002b; Jug et al., 2021). SOBI is robust in low SNRs (Belouchrani et al., 1993; Cardoso and Souloumiac, 1996; Tang et al., 2002b; Wam et al., 2004; Goshvarpour et al., 2016; Zangeneh Soroush, 2021). Since SOBI is iterative, it is found to be one of the fastest algorithms, compared to previous methods such as ICA, compared with other BSS methods (Wam et al., 2004). It should be mentioned that recently other versions of BSS methods such as canonical correlation analysis (CCA) have been introduced and are faster than SOBI; however, their performance in EEG artifact removal is not higher in all cases. Compared to ICA, SOBI relies on a second-order statistical analysis of signals while ICA is based on higher-order statistics, which means ICA is more time-consuming, complex, and laborious (Zangeneh Soroush, 2021). These features suggest that the SOBI method of source separation is effective (Belouchrani et al., 1993; Cardoso and Souloumiac, 1996; Goshvarpour et al., 2016). These characteristics motivated us to use SOBI in this study.

### 2.2 Phase space and angle space reconstruction

Phase space reconstruction (PSR), has become a useful tool in nonlinear signal processing in numerous studies (Zangeneh Soroush et al., 2017; Zangeneh Soroush et al., 2018a; Zangeneh Soroush et al., 2018b; Zangeneh Soroush et al., 2018c; Zangeneh Soroush et al., 2018d; Zangeneh Soroush et al., 2019a; Zangeneh Soroush et al., 2019b; Zangeneh Soroush et al., 2020; Zangeneh Soroush, 2021). This robust analysis introduces a new transformation and several characteristics of a given signal by retaining signals’ magnitude and phase information. This motivated us to study these characteristics with the goal of automated source identification. Phase space includes state vectors describing the signal. There are several ways to reconstruct the phase space of a signal. Reviewing previous studies, we turn to the most common method, time delay embedding (Zangeneh Soroush et al., 2018b; Zangeneh Soroush et al., 2019b). Suppose that v(t) is a signal with K time samples. We can reconstruct K−d+1 vectors in the phase space as:

V(i)=[v(i+T)  v(i+2τ) …  v(i+(d−1)τ)]             i=1.2.….K−(d−1) (3)

Where d and τ are the embedding dimension and time delay, respectively. d and τ are important parameters while reconstructing phase space (Zangeneh Soroush et al., 2018a; Zangeneh Soroush et al., 2019a). Based on previous studies, the value of d is chosen as two, and τ is 0.2-times the standard deviation of the signal (Zangeneh Soroush et al., 2017; Zangeneh Soroush et al., 2018a; Zangeneh Soroush et al., 2018b).

### 2.3 Angle space reconstruction

Having reconstructed the phase space of the signal, we consider the angle between each three points (in row) as a geometrical characteristic of the phase space. In other words, each line connecting points in the phase space is considered a vector. The angles between vectors and also the vector length are calculated in order to transform the phase space into a new state space called angle space (AS) (Zangeneh Soroush et al., 2017; Zangeneh Soroush et al., 2018a; Zangeneh Soroush et al., 2018b; Zangeneh Soroush et al., 2018c; Zangeneh Soroush et al., 2018d; Zangeneh Soroush et al., 2019a; Zangeneh Soroush et al., 2019b; Zangeneh Soroush et al., 2020; Zangeneh Soroush, 2021). Angle space reconstruction leads to two sequences of angle values (AV) and vector lengths (VL) which contain valuable information about the underlying signal. Vector length is set to the unit for all points in AS to achieve AP. Therefore, we suppose the vector length is equal to one, and all angle values are transferred to the X-Y coordination on the unit circle to study angle space and its dynamics. Here, we just consider the angle values on the unit circle ( r=1 ) called angle plot (AP). It can be considered a new representation of a signal (Zangeneh Soroush et al., 2018b). Different features are defined and then extracted from this new representation.

### 2.4 Feature extraction based on AP and poincare planes

#### 2.4.1 Poincare plane

Poincare sections are considered a geometrical description of state space. Poincare planes (PPs) are defined in one dimensional less than the corresponding state space. PPs enable us to analyze signal trajectories and transitions. Choosing appropriate PPs is of a great deal of importance. Thanks to suitable PPs, maximum information about system dynamics and changes is transferred and also down-sampled (Sharma et al., 2015). Having reviewed previous studies, we came to a conclusion to employ five suggested PPs (Takens et al., 1981; Acharya et al., 2012; Lee et al., 2014; Fang et al., 2015; Sharma et al., 2015; Sadeghi Bajestani et al., 2017). We call these five sections PP1 to PP5. Table 1 represents the Poincare planes we used in this study and the abbreviations.

**TABLE 1:** Poincare planes used in this study.

#	Abbreviation	Description
1	PP1	X axis
2	PP2	Y axis
3	PP3	Diagonal line (first and third quadrant bisector)
4	PP4	Perpendicular to diagonal line (second and fourth quadrant bisector)
5	PP5	Circular plane with the radius of r=0.001

As mentioned before, features are extracted based on AP and the proposed Poincare planes. Statistical features containing mean, variance, skewness, and kurtosis are extracted from AP. Features employed for source identification are explained in Table 2. Statistical features including average, variance, skewness, and kurtosis of the angle values are extracted. The number of intersections (for each PP) is also considered a feature.

**TABLE 2:** Extracted features from AP and PPs for source identification.

#	Feature description	Abbreviation
1	Average of angle values	AveAP
2	Variance of angle values	VaAP
3	Skewness of angle values	SkAP
4	Kurtosis of angle values	KuAP
5	Median of angle values	MeAP
6	Shannon’s entropy of angle values	ShAP
7	Length of the angle time series	LeAP
8	Number of intersections with PP1	$NPP_{1}$
9	Number of intersections with PP2	$NPP_{2}$
10	Number of intersections with PP3	$NPP_{3}$
11	Number of intersections with PP4	$NPP_{4}$
12	Number of intersections with PP5	$NPP_{5}$

Results show that these features are significant. These features are extracted from each source. Source Identification is performed based on these features.

### 2.5 Classification

K-nearest-neighbor (KNN), Naïve Bayes, support vector machine (SVM), and multi-layer-perceptron (MLP) are four basic and standard classifiers that generate immense interest in numerous studies in different fields. They are employed in this paper to have a more comprehensive study. We used 10-fold cross-validation to evaluate our classifiers using average EEG source classification accuracy. KNN classifies unknown input data according to the K closest training samples. The parameter K is the main factor in this classifier. We took a trial-and-error approach to determine the best value for K, which is 20 in this study. KNN is very effective while samples have spherical distribution is the feature space because it classifies samples based on the distances and nearest neighbors. MLP is a fully-connected neural network with input, hidden, and output layers. Each layer consists of several neurons connected *via* weights, which are determined through the learning process. The number of neurons in the input layer is equal to the number of features, while the number of neurons in the hidden and output layer is variable and should be defined with respect to the application and data. We took a trial-and-error approach to determine the number of neurons in the only hidden layer. It was determined as 10 in this study. The number of neurons in the output layer was equal to the number of classes. We employed the Levenberg-Marquart learning algorithm to train our MLP classifier and adjust its weights. Naïve Bayes is the third classifier we employed in this project. We take advantage of the Bayesian classifier’s properties in minimizing the classification error based on probability density functions of training samples. Bayes provides us with a decision boundary in the probability density functions to classify an unknown given test data. SVM uses the training data to identify support vectors which are the closest training samples from different classes and can determine the decision boundary. We used the original SVM with a linear kernel (decision boundary). SVM has been approved as an effective classifier since it is less affected by the size of the training data, compared to other classifiers such as MLP. These classifiers are explained precisely in other works like (Cardoso and Souloumiac, 1996; Goshvarpour et al., 2016; Zangeneh Soroush et al., 2017; Zangeneh Soroush et al., 2018a; Zangeneh Soroush et al., 2018b; Zangeneh Soroush et al., 2018c; Zangeneh Soroush et al., 2018d; Zangeneh Soroush et al., 2019a; Zangeneh Soroush et al., 2019b; Zangeneh Soroush et al., 2020; Zangeneh Soroush, 2021), so we avoid reviewing them here. Since source recognition is part of this study, we report classification accuracy for these conventional classifiers to compare the results.

### 2.6 Wavelet-based artifact removal

**FIGURE 2:**
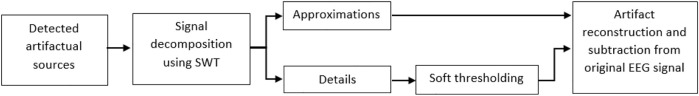
The block diagram for the proposed artifact elimination method based on one-level SWT with “haar” wavelet basis function as its wide usage in EEG preprocessing applications.

Different algorithms can be taken into account to remove artifacts. One can set artifactual components to zero, which is not very practical since neural information is very possible to leak into these components. So, ignoring all artifactual sources might lead to information loss. Although this approach seems to be very simple, it leads to significant distortion in reconstructed EEGs. On the other hand, a well-known algorithm to suppress artifacts is decomposing artifactual components by wavelet transform. Decomposed sub-bands are denoised by thresholding (Yang et al., 2018). Several studies have suggested wavelets including discrete wavelet transform (DWT), continuous wavelet transform (CWT), or stationary wavelet transform (SWT) with the aim of artifact elimination (Castellanos and Makarov, 2006; Hoffmann and Falkenstein, 2008; Hamaneh et al., 2014). As it is stated in (Coifman and Donoho, 1994; Hoffmann and Falkenstein, 2008; Ng and Raveendran, 2009; Yang et al., 2018), SWT is superior to DWT and CWT in removing biological artifacts. Additionally, SWT is translation-invariant, suggesting its superiority to DWT while removing biological artifacts. According to the results in (Hoffmann and Falkenstein, 2008), we employ SWT to denoise detected artifactual components. Figure 2 represents the block diagram of the suggested artifact removal approach using SWT.

We decided to use Haar wavelet because of its advantages in comparison with other wavelet basis functions, five levels of decompositions, and soft thresholding, as suggested in (Hoffmann and Falkenstein, 2008). Wavelet analysis results in obtaining approximations and details corresponding to strong artifacts and cerebral information, respectively. Artifactual sources are decomposed, and sub-bands are taken into the thresholding step since, in this application, approximations correspond to artifacts, and obviously, details pertaining to cerebral activity. So we apply soft thresholding to remove small values in details. Inverse SWT is applied to approximation and thresholded details to achieve artifacts-only signals. Then the reconstructed artifacts are subtracted from the original signal to have clean EEGs. By thresholding, small values of leaked EEGs would be removed, and consequently, artifact-only components could be reconstructed, projected back to EEG channels, and then subtracted from EEG data (Prado et al., 2019; Bui and Chen, 1998; Romero et al., 2008). The proposed denoising algorithm is fast, and simple. Like (Hoffmann and Falkenstein, 2008), we choose five levels of decomposition, and the MATLAB function ddencmp computes the global threshold.

### 2.7 Source identification and artifact removal performance measures

Although artifact removal methods are mainly evaluated based on different criteria, the evaluation procedure has always been problematic because there is no universal or general quantitative criterion (Yang et al., 2018). Method’s effectiveness can be analyzed through visual inspection by experts, which is subjective and not standard. We consider both subjective and objective metrics in this study. Experts label real and synthesized signals and also extracted sources. So classification performance is the first performance measure. In addition, artifactual sources are suppressed and then “clean” EEG is reconstructed. Therefore, we can define other metrics to evaluate the proposed artifact removal method. Based on the previous studies (Sanei and Chambers, 2007; Islam et al., 2016; Romo Vázquez et al., 2012; Klemm et al., 2009; Croft and Barry, 2000; Lagerlund et al., 1997; Li et al., 2006; Senthilkumar, 2008; Mumtaz et al., 2021; Yang et al., 2018; Cao et al., 2015; Rahman et al., 2015; Sai et al., 2018; Rodr´ıguez-Berm´udez and Garcia-Laencina, 2015; Mahajan and Morshed, 2015; Shoker et al., 2005; Jung et al., 2000; Vorobyov and Cichocki, 2002; Belouchrani et al., 1997; Delorme and Makeig, 2004), some common measures are introduced as the evaluation criteria in this study.

#### 2.7.1 Classification performance

Classification accuracy is defined based on the proportion of the number of correctly classified test samples and the number of total test samples. Employing 10-fold cross-validation in this study, average classification performance (ACP), the mean classification accuracy, is calculated and reported for each classifier.

#### 2.7.2 Temporal and spectral relative root-mean-square and mean-absolute errors

Artifact removal systems can be evaluated using the time domain’s relative root-mean-square error (RRMSE). Several studies consider this factor an artifact suppression evaluation parameter (Makinen et al., 2005; Onton and Makeig, 2006; Fang et al., 2015). RRMSE is defined in the time domain as below:

$$RRMSE\left(X\right)=\frac{RMS\left(X-\hat{X}\right)}{RMS\left(X\right)}$$ (4)

$$RMS\left(X\right)=\;\sqrt{\frac{1}{N.K}\overset{N}{\underset{i=1}{\sum }}\overset{K}{\underset{j=1}{\sum }}X^{2}\left(i.j\right)\;\;}$$ (5)

Where X and $\hat{X}$ are contaminated (i.e., before artifact removal) and reconstructed (i.e., after artifact removal) EEGs, respectively. It can be easily expanded to the frequency domain in order to estimate relative root-mean-square error using power spectral density (PSD), which leads to another measure (i.e., $RRMSE_{PSD}$ ) described as following:

$$RRMSE_{PSD}\left(X\right)=\frac{RMS\left(PSD_{X}-PSD_{\hat{X}}\right)}{RMS\left(PSD_{X}\right)}$$ (6)

Whit $PSD_{X}$ and $PSD_{\hat{X}}$ indicating PSD of the clean EEG and denoised EEGs, respectively. This measure enables us to analyze the results and evaluate the method with respect to the spectral properties of EEGs. We also used mean absolute error (MAE) in power spectral density which is called RRMAE_PSD_ to evaluate our proposed method using Eq. 7. Although RRMSE_PSD_ can provide us with a practical measure, RRMAE_PSD_ can also be used as it measures the difference between the contaminated signals and the reconstructed ones in the frequency domain as below:

$$RRMAE_{PSD}\left(X\right)=\;\;MAE\left(PSD_{X}-PSD_{\hat{X}}\right)$$ (7)

#### 2.7.3 Average correlation coefficient

Correlation coefficients (CCs) between original EEGs (not corrupted) and reconstructed ones are valuable metrics to evaluate how effectively the proposed artifact removal method can eliminate artifacts. For simulated and semi-simulated signals, original EEGs and artifacts are available. Therefore average correlation coefficient (ACC) could be an evaluation measure. The third criterion in this study is the ACCs overall reconstructed EEG channels with respect to the corresponding original EEG channels (Chen et al., 2017).

#### 2.7.4 Average mutual information

Correlation coefficients cannot fully describe the similarity between two signals. Therefore we decided to employ mutual information (MI) as an index to evaluate the similarity of signal dynamics between original EEGs and reconstructed ones. As another evaluation parameter, average mutual information (AMI) values are computed over all channels. Several studies have applied AMI to quantify their methods (Makinen et al., 2005; Onton and Makeig, 2006; Hayashi et al., 2015; Sayed et al., 2017; Sharif and Homayoun Jafari, 2017). MI is computed in Eq. 8 as

$$MI=\;\overset{+\infty }{\underset{-\infty }{\int }}\overset{+\infty }{\underset{-\infty }{\int }}p\left(X.\hat{X}\right)\log\left(\frac{p\left(X.\hat{X}\right)}{p\left(X\right)p\left(\hat{X}\right)}\right)dX\;d\hat{X}$$ (8)

where $p\left(X,\hat{X}\right)$ is the joint probability density function of X (i.e., original EEG) and $\hat{X}$ (i.e., reconstructed EEG after artifact removal). p(X) and $p\left(\hat{X}\right)$ represent marginal probability density functions of X and $\hat{X}$ , respectively. Since AMI indicates the relevance between two signals, it is clear that the larger the AMI is, the more effective the proposed method will be (Hoffmann and Falkenstein, 2008).

### 2.8 Power spectral density in truncated frequency bands

As one of the previous studies (Fang et al., 2015) proposed a criterion using the PSD in frequency bands 1–3, 3–20 Hz and above 20 Hz, we also decided to introduce a new performance measure based on that. It is considered that artifacts concentrate in two frequency bands which are 1–4 Hz and above 30 Hz. It is also assumed that brain activity is within 4–30 Hz. So PSD for artifacts and EEGs can be computed before and after artifact removal. As it is suggested in (Fang et al., 2015), there is a trade-off when estimating the following measure:

$$PSD_{tr}=\frac{RMS(PSD_{EEG}-\hat{PSD_{EEG})}}{RMS(PSD_{1-4}-\hat{PSD_{1-4})}+RMS(PSD_{above\;30}-\hat{PSD_{above\;30})}}$$ (9)

Where $PSD_{1-4}$ shows the spectrum of EEG in 1–4 Hz $PSD_{above\;30}$ is related to the spectrum of EEG in the frequency band above 30 Hz $PSD_{EEG}$ indicates the signal spectrum between 4 and 30 Hz. PSDs without a tilde sign refer to contaminated EEG and those with the tilde sign indicate EEG after artifact removal. The smaller this performance parameter is, the more effective the proposed method will be. Small values for this measure is possible with a small numerator which suggests good performance in preserving EEGs and large denominator which shows successful artifact suppression.

### 2.9 Database

#### 2.9.1 Simulated data

**FIGURE 3:**
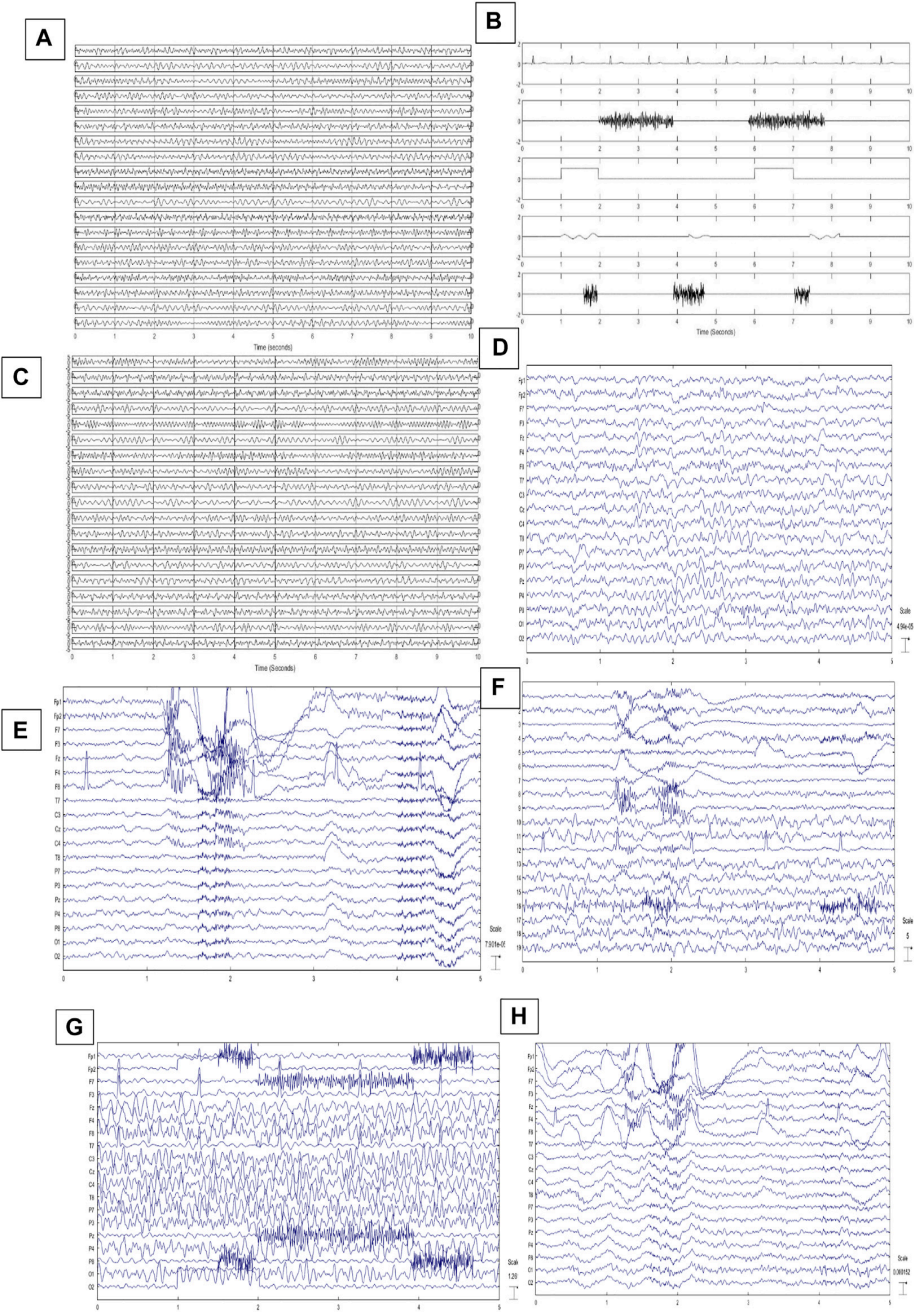
**(A)** 10-s illustration of PSEEGs, **(B)** Synthesized artifacts, **(C)** SCEEGs by projecting artifacts at SNR=0.5db , **(D)** An example for recorded PREEG for 5 s, **(E)** RCEEGs of a subject for 5 s, **(F)** extracted sources using SOBI algorithm, **(G)** Illustration of 19-channel SSCEEG1s at SNR=0.5 , **(H)** An illustration of SSCEEG2s at SNR=0.5db .

Generating simulated EEGs is introduced in (Makinen et al., 2005) based on the phase-resetting theory. According to (Yeung et al., 2007), EEGs can be reconstructed by adding four sinusoids with randomly chosen frequencies varying from 4 to 30 Hz. Frequency values are selected independently and randomly to synthesize EEGs. So we can easily construct pure-simulated EEGs (PSEEG) by adding four sinusoids. This method is also completely explained in (Chen et al., 2017). To reconstruct a 1-min single-channel signal, thirty 2-s segments are generated and concatenated together. Nineteen channels of EEG and also modeled artifacts are reconstructed in this way. Figure 3A shows one example for PSEEGs.

All signals are recorded or synthesized with a sampling frequency of 256 Hz. We model EEGs and artifacts as below:

• EEG: summation of four sinɛ functions at random frequencies in the range of 4–30 Hz (Yeung et al., 2007),
• ECG: can be reconstructed by Auto-Regressive (AR) modeling. Parameters are estimated using real ECG recordings. Then, artificial ECGs are reconstructed utilizing AR modeling. We chose AR order as 12 based on Akaike Information Criterion (AIC) and Bayes Information Criterion (BIC) (Rissanen, 1999; Padmavathi and Sri Ramakrishna, 2015) (the average order was 11.6 with a standard deviation of 1.1),
• EMG: temporal muscle activity is modeled by filtering (FIR) random noise in the frequency range of 20–60 Hz (Delorme et al., 2007; Chen et al., 2017; Bai et al., 2016),
• EOG: eye movement is modeled through low-frequency square pulses with the frequency of 0.2 Hz (Hoffmann and Falkenstein, 2008; Chen et al., 2017; Bai et al., 2016),
• Eye blinking: we synthesize eye blinking artifact using random noise band-pass filtered between 1 and 3 Hz (Delorme et al., 2007).
• White noise: an unfiltered white noise is employed as an artifact as well.

All five generated artifacts are synthesized in 2-s segments. We generated artifacts in segments with random lengths varying from 500 m to 2 s. In other words, a 2-s window consists of an artifact based on a random selection. Each modeled artifact is projected to all 19 channels *via* a random transformation matrix containing at least ten non-zero random entries and then summed with 19-channel PSEEGs to artificially generate simulated contaminated EEGs (SCEEG). The intensity of artifacts and corresponding channels are randomly selected according to the normal uniform distribution. Based on (Fang et al., 2015), artifacts can be added to PSEEGs at different levels of signal-to-noise ratio (SNR). Eq. 3 represents the summation of artifacts and EEGs.

$$X_{C}=X_{P}+\lambda .X_{ART}$$ (10)

Where λ indicates the artifact intensity and totally affects SNR. $X_{C}$ shows the corrupted 19-channel EEGs. $X_{P}$ and $X_{ART}$ demonstrate pure EEGs and modeled 19-channel artifacts, respectively. SNR is defined based on Eqs 4, 5.

$$SNR=\frac{RMS\left(X_{P}\right)}{RMS\left(\lambda .X_{ART}\right)}$$ (11)

$$RMS\left(X\right)=\;\sqrt{\frac{1}{N.K}\overset{N}{\underset{i=1}{\sum }}\overset{K}{\underset{j=1}{\sum }}X^{2}\left(i.j\right)\;\;}$$ (12)

Where N is the number of channels and K shows time samples. For more information about simulated-contaminated EEG generation, refer to (Chen et al., 2017). Figures 3B,C illustrate one example of generated artifacts and CSEEGs.

#### 2.9.2 Real data

The EEG signals are recorded from twenty individuals (10 males). 19 Ag/Ag-Cl electrodes, according to the 10–20 international standards, are placed on each subject’s scalp. EEGs are acquired and sampled at 256 Hz for 1 min in each trial. Each individual participates in 20 separate trials. EEGs are recorded while normal subjects are sitting in a comfortable fashion with their eyes open (Lawhern et al., 2012). In the first ten trials for each individual, subjects are acquired not to move their head, jaw, or eyebrows. Also, eye blinking or movements are visually inspected and not considered in the database. Recorded EEGs are filtered through conventional filtering methods such as bandpass (4–60 Hz) and 50-Hz notch filters based on previous studies like (Delorme et al., 2007; Lawhern et al., 2012) in order to have clean EEGs with no artifacts. Three expert clinicians controlled the recording process and justified clean EEGs. The first ten trials are called pure real EEGs (PREEG). We have PREEGs in 19 channels and ten 1-min trials for twenty subjects. A sample of PREEGs is represented in Figure 3D.

In the next phase, subjects are asked to blink both eyes (without squinting) and randomly move their eyes (vertically and horizontally) and eyebrows for 1 min in each trial. Subjects are left free to blink or move their eyes or eyebrows in their natural manner. Movements are performed in separated and different trials. Eye blinking, eye movement, and moving eyebrows are performed in the second ten trials. Subjects are previously informed not to move or tilt their head. Vertical and horizontal EOGs and also ECG are captured in both phases with the aim of helping clinicians while recognizing sources. It should be noted that only 19 contaminated EEG channels are used in further analyses, and other signals are recorded due to getting monitored by clinicians. EEGs are filtered by conventional bandpass and notch filters. Subjects are controlled visually while recording signals and movements are recorded in time course. In this phase, individuals participate in ten trials to have real contaminated EEGs (RCEEG). Then RCEEGs and extracted sources *via* the SOBI algorithm are analyzed by clinicians to label sources. Figures 3E,F show an illustration of RCEEGs and extracted sources through the SOBI algorithm respectively. Experts are inquired to put each source in one category from all six groups containing EEG, ECG, EMG, EOG, eye blink, and white noise.

Since artifacts including ECG, eye blinking, EMG, EOG, and white noise are significant and prominent in most BCI applications, our focus in this study is on these common artifacts, and other artifacts like head movement, power-line noise, and electrical shift are ignored (Delorme et al., 2007; Yang et al., 2018). Expert clinicians, including three neurophysiologists, are informed to control the experiments and label extracted sources based on the mentioned artifacts. In this phase, we have RCEEGs in 19 labeled sources and ten 1-min trials for each of the twenty subjects.

#### 2.9.3 Semi-simulated data

Two semi-simulated datasets are provided to study the proposed method. In the first dataset, EEGs are taken from PREEGs, and generated artifacts are randomly projected and summed at different SNR values. Then extracted sources are identified by experts. The first set of semi-simulated contaminated EEGs (SSCEEG1) is reconstructed using Eq. 9 at different SNR values. Figure 3G illustrates one typical 5-s SSCEEG at SNR=0.5 . Synthetic artifacts explained in the previous sub-section are projected with varying intensities and then added to pure EEG recordings.

For the second semi-simulated dataset, we use EEGs which are randomly selected from PREEGs. We also recorded EEGs from other 20 individuals asked to move their eyebrows, blink both eyes and move their eyes horizontally and vertically. Other types of mentioned artifacts like ECG or white noise are seen in the recordings. Then artifacts are extracted *via* FastICA algorithm and identified by experts. Extracted artifacts are just considered and then projected back to PREEGs for further analyses. Figure 3H illustrates one example for SSCEEG2. In this approach, we have 200 PREEGs (20 subjects, 10 trials) from the first group of participants and 200 samples which are recorded from the second group of individuals. Extracted artifacts are randomly selected and projected to PREEGs at different SNR s to reconstruct the second set of semi-simulated contaminated EEGs (SSCEEG2). We have the second type of semi-simulated signals containing real artifacts and real EEGs. Figure 3H shows one example for a 5-s semi-simulated contaminated EEG.

## 3 Results

As mentioned before, four different datasets (SCEEG, RCEEG, SSCEEG1, and SSCEEG2) are provided in this study. 200 different 19-channel simulations or recordings are considered for each dataset. We apply the proposed method to different signal lengths to analyze the results more comprehensively. 10, 30, and 60-s windows are considered for signals in this study. Table 3 represents the average and standard deviation of classification accuracy for all datasets. ACP shows the average accuracy while classifying samples in each dataset and at different signal lengths. In Table 3, the employed MLP has just one hidden layer, and the number of neurons in the hidden layer was determined through a trial-and-error process equal to 10. We used this process to find the best parameter, which results in the highest classification performance in the training phase. For the KNN algorithm, we conducted the same procedure and determined the parameter K equal to 20.

**TABLE 3:** Average and standard deviation of classification accuracy in the 6-class scenario using all of the datasets, including simulated, semi-simulated and real EEGs.

Length (s)	Classifier	SCEEG	SSCEEG1	SSCEEG2	RCEEG
10	MLP	75.72±6.55	76.64±5.52	76.16±6.33	75.62±6.51
	KNN (K = 20)	76.53±7.41	76.17±6.09	76.32±7.11	75.85±7.16
	Bayes	77.04±8.69	76.39±5.36	76.22±6.27	75.49±7.23
	Ensemble	78.12±5.74	79.06±5.87	78.93±5.85	78.86±5.95
30	MLP	76.49±7.93	75.84±6.16	75.33±7.41	75.37±7.79
	KNN (K = 20)	76.31±7.01	75.92±6.09	75.87±6.71	75.41±8.23
	Bayes	76.17±7.39	75.78±7.04	75.63±8.08	75.49±6.23
	Ensemble	78.01±7.68	78.17±7.98	77.93±7.15	78.86±5.95
60	MLP	77.06±6.55	75.64±6.52	75.16±6.33	75.62±7.51
	KNN (K = 20)	76.53±6.41	75.17±5.09	75.32±5.35	75.85±6.16
	Bayes	76.04±8.69	75.39±7.44	75.22±7.29	75.49±7.23
	Ensemble	78.63±7.58	77.63±6.57	78.06±6.32	76.93±6.04

Classification results suggest that the proposed features and classifiers are effective to identify artifacts. Six groups containing EEG, EMG, ECG, EOG, eye blinking, and white noise are considered in the classification. All accuracy results are quite high and in the same range. It shows that the signal length is not objective in the proposed method. That is to say that results for real EEGs are really similar to that of simulated and semi-simulated ones. It can be easily seen that the ensemble of three classifiers outperforms each of them. Therefore, we apply the ensemble of classifiers in further analyses to evaluate the proposed method. For simplicity, results are given in the three following subsections to make a better comparison. We bring the results just for 10 s EEGs in the following sections for the sake of space. Since, in most studies, it is of great importance to classify artifacts and brain activities, we also decide to classify components into two classes containing neural and artifactual components. Table 3 illustrates the classification results when six classes of artifacts are going to be recognized, and Table 4 reports the classification performance while classifying EEG components into two classes, including artifactual and “clean.” In other words, in Table 3, we aim to examine how effective our proposed method is in recognizing artifact types, while in Table 4, we report how successfully our method can detect artifactual EEG components estimated by SOBI. Three aforementioned classifiers and the mixture classification model are employed in both scenarios, including 6-class and 2-class scenarios. Clean components are assumed and named as neural components as we suppose they just contain neural information.

**TABLE 4:** Classification results (in the 2-class scenario) for recognizing neural and artifactual components. Acc, Sen, Spc and Per present classification accuracy, sensitivity, specificity and precision, respectively. Ensemble represents the mixture of classifiers.

Length (s)	Classifier		SCEEG	SSCEEG1	SSCEEG2	RCEEG
10	MLP	Acc	95.54 ± 2.96	96.09 ± 2.28	94.92 ± 3.88	94.17 ± 2.15
		Sen	96.83 ± 3.02	96.11 ± 3.64	95.07 ± 2.61	94.02 ± 3.98
		Spc	95.74 ± 3.04	94.14 ± 3.02	94.56 ± 2.99	95.84 ± 1.78
		Per	95.86 ± 3.67	95.08 ± 2.99	94.21 ± 1.81	94.47 ± 3.17
	KNN (K = 20)	Acc	95.32 ± 3.39	94.79 ± 3.04	95.84 ± 3.45	96.00 ± 2.06
		Sen	96.69 ± 3.27	95.89 ± 1.95	94.11 ± 3.39	95.96 ± 2.81
		Spc	95.57 ± 2.99	94.99 ± 2.92	95.10 ± 3.00	95.52 ± 3.08
		Per	95.34 ± 3.26	96.53 ± 3.55	94.46 ± 2.94	94.98 ± 3.08
	Bayes	Acc	96.58 ± 3.05	94.23 ± 3.07	95.30 ± 2.12	94.97 ± 3.26
		Sen	97.77 ± 2.83	95.37 ± 3.02	94.40 ± 3.38	94.20 ± 2.95
		Spc	96.65 ± 3.16	94.77 ± 2.99	95.49 ± 2.08	96.02 ± 3.92
		Per	97.03 ± 3.12	96.12 ± 2.98	95.74 ± 3.04	94.87 ± 2.87
	Ensemble	Acc	98.73 ± 3.01	97.91 ± 3.08	97.71 ± 3.66	96.29 ± 3.01
		Sen	97.94 ± 3.30	98.03 ± 3.39	96.81 ± 2.75	96.35 ± 3.50
		Spc	97.71 ± 3.12	97.55 ± 2.99	96.86 ± 3.23	97.78 ± 3.03
		Per	98.80 ± 3.01	98.10 ± 2.98	97.16 ± 3.39	97.41 ± 3.38
30	MLP	Acc	94.88 ± 3.34	96.54 ± 2.36	96.35 ± 3.46	95.71 ± 3.22
		Sen	96.49 ± 2.87	95.09 ± 1.95	94.93 ± 2.39	94.15 ± 2.71
		Spc	96.41 ± 2.90	93.51 ± 3.71	95.96 ± 3.11	95.88 ± 2.67
		Per	96.42 ± 2.98	94.26 ± 2.21	95.12 ± 3.71	97.53 ± 3.73
	KNN (K = 20)	Acc	95.67 ± 3.11	94.94 ± 2.97	96.44 ± 3.03	96.66 ± 2.58
		Sen	93.79 ± 2.72	97.35 ± 3.30	95.04 ± 3.09	95.31 ± 3.07
		Spc	95.72 ± 2.74	94.38 ± 2.20	94.80 ± 3.51	95.74 ± 3.24
		Per	96.63 ± 2.53	95.75 ± 3.07	95.79 ± 3.57	94.13 ± 2.27
	Bayes	Acc	95.49 ± 2.84	94.81 ± 2.58	97.91 ± 2.84	94.82 ± 2.85
		Sen	96.03 ± 2.80	95.89 ± 3.04	95.83 ± 2.93	95.79 ± 1.97
		Spc	95.73 ± 3.48	94.24 ± 3.12	96.38 ± 3.54	95.67 ± 3.26
		Per	94.70 ± 2.76	95.60 ± 3.24	94.94 ± 2.62	94.67 ± 2.74
	Ensemble	Acc	97.29 ± 2.65	96.58 ± 3.26	97.53 ± 2.78	96.55 ± 2.81
		Sen	96.21 ± 3.16	97.49 ± 3.92	96.73 ± 2.08	97.33 ± 2.96
		Spc	97.89 ± 3.75	96.82 ± 2.82	96.10 ± 3.64	97.39 ± 3.29
		Per	96.85 ± 2.82	97.80 ± 2.64	97.72 ± 3.18	96.45 ± 2.21
60	MLP	Acc	93.93 ± 2.36	96.42 ± 3.09	95.7 ± 2.89	96.87 ± 3.33
		Sen	94.19 ± 2.95	95.29 ± 3.01	92.95 ± 2.95	95.18 ± 3.25
		Spc	92.06 ± 2.06	95.20 ± 2.96	94.65 ± 3.01	94.52 ± 2.94
		Per	96.44 ± 3.04	96.59 ± 3.49	94.18 ± 2.88	95.86 ± 3.07
	KNN (K = 20)	Acc	95.33 ± 2.92	94.20 ± 3.25	94.42 ± 3.13	94.64 ± 2.82
		Sen	94.25 ± 3.19	95.70 ± 3.23	95.51 ± 3.17	95.46 ± 2.93
		Spc	96.37 ± 3.20	95.84 ± 3.80	95.28 ± 2.29	94.15 ± 3.31
		Per	93.29 ± 2.42	94.76 ± 3.63	95.03 ± 2.83	94.67 ± 2.73
	Bayes	Acc	94.90 ± 2.17	95.22 ± 3.15	95.67 ± 2.35	95.55 ± 3.19
		Sen	94.76 ± 2.61	95.83 ± 2.34	96.13 ± 2.76	96.04 ± 3.82
		Spc	95.32 ± 2.52	94.85 ± 3.57	95.35 ± 2.97	95.88 ± 2.34
		Per	95.31 ± 2.79	95.10 ± 2.88	94.70 ± 3.43	96.26 ± 2.69
	Ensemble	Acc	97.14 ± 3.01	96.72 ± 3.19	96.02 ± 3.06	96.66 ± 3.11
		Sen	98.23 ± 2.37	97.59 ± 2.83	96.74 ± 2.38	97.93 ± 3.05
		Spc	96.84 ± 2.69	96.33 ± 3.27	97.25 ± 1.98	96.80 ± 2.83
		Per	96.63 ± 3.15	95.19 ± 2.92	96.71 ± 2.43	96.78 ± 2.95

In this table Acc, Sen, Spc, and Per indicate classification accuracy, sensitivity, specificity and precision respectively. These measures suggest how successful our suggested approach is. Taking a close look at the two tables for 6-class and binary artifact detection, we come to the conclusion that our classification performance is much higher in the binary classification scenario when we just need to determine whether the given EEG component is artifactual or “clean.” One reason could be similarity between extracted features in a 6-class scenario that make the classification problem even more difficult. For example, artifacts associated with EOG and electrode displacement may share quite similar dynamics in the angle plot and can result in similar features, making the classification step more difficult. This issue will be explored later in the discussion section. Moreover, in classification problems, classification accuracy almost pertains to the number of classes suggesting that the more classes exist, the more challenging the classification task would be. Additionally, one can infer that our proposed approach results in features which are almost similar in some artifact classes and make the two bigger classes, including clean components and artifactual ones. This might be assumed as a disadvantage of our proposed method; however, since our final results which are discussed later, are acceptable and comparable to recent studies, we can still consider our suggested approach quite effective in EEG artifact removal. In addition, in most practical applications, we may just need to know if the given component is contaminated or not, and their origins are not of great importance.

Detected components as artifacts are fed into SWT-based artifact removal. Considering Acc and Sen measures, it is evident that the ensemble of the classifiers is more efficient and successful compared to each classifier alone.

To analyze the results more completely, we decided to report the features’ average and standard deviation in Table 5. So, all artifact components in all datasets are put in one group and sources related to brain activity in the other group. We performed a t-test analysis to examine how effective our proposed features are. Average and standard deviation values are reported for both artifactual and clean classes. It should be noted that all EEG signals from real, semi-simulated, and simulated are used in this analysis. EEG components were divided into two classes, including “Artifactual” and “Clean.” Most significant features whose *p*-values are lower than 0.05 are reported in Table 5. All components are normalized to the range of [−1 1] before features’ statistics estimation in order to have the same amplitude range for all components. Average and standard deviation values are computed for all EEGs over each extracted feature. T-test is also carried out to investigate the level of significance for each proposed feature. The most significant features are highlighted. Considering Table 5, we can easily find out that most proposed features are significant enough to be included in the next step, where classification models are going to be trained using these features. As it is clear, all features related to Poincare planes have a *p*-value less than 0.05. It shows the importance of nonlinear analysis of signal dynamics, and Poincare planes are able to describe the characteristics of the components. Besides, 2-class classification is carried out over all normalized components. Features whose *p*-value is less than 0.05 (are highlighted in Table 5) are selected for each component, and then classification is carried out. Table 6 represents the classification results for the artifactual and clean EEG component recognition. The most significant features, selected by t-test and reported in Table 5, are used in this classification.

**TABLE 5:** Average and standard deviation values for the proposed features. T-test was performed for the proposed features, and *p*-values were reported.

#	Feature abbreviation	Ave ± std in the brain activity class	Ave ± std in the artifact class	*p*-value
1	AveAP	37.46 ± 5.17	40.02 ± 4.92	0.0657
2	VaAP	43.81 ± 12.57	49.75 ± 10.02	0.0789
3	SkAP	0.94 ± 0.18	0.69 ± 0.39	0.0491
4	KuAP	3.67 ± 0.94	3.01 ± 0.64	0.0476
5	MeAP	73.31 ± 5.58	60.15 ± 6.70	0.0581
6	ShAP	982,312.37 ± 37.93	977,375.96 ± 42.12	0.0464
7	LeAP	3,217.03 ± 10.56	3,102.66 ± 9.36	0.0812
8	$NPP_{1}$	312.87 ± 8.56	198.56 ± 9.03	0.0299
9	$NPP_{2}$	367.35 ± 10.11	245.73 ± 9.26	0.0438
10	$NPP_{3}$	312.48 ± 11.78	456.87 ± 10.49	0.0327
11	$NPP_{4}$	327.87 ± 12.07	423.87 ± 9.06	0.0492
12	$NPP_{5}$	189.58 ± 7.92	163.44 ± 6.65	0.0413

**TABLE 6:** Classification performance in the 2-class scenario using the most significant features from Table 5 for the normalized EEG components over all samples from simulated, semi-simulated, and real ones.

Classifier		10 s	30 s	60 s
MLP	Acc	97.63 ± 1.57	96.36 ± 1.32	96.43 ± 1.38
	Sen	97.22 ± 1.32	96.27 ± 1.61	96.46 ± 1.51
	Spc	97.53 ± 1.38	97.29 ± 1.12	95.67 ± 1.75
	Per	96.37 ± 1.24	96.88 ± 1.26	96.58 ± 1.36
KNN (K = 20)	Acc	96.94 ± 1.49	97.42 ± 1.49	96.31 ± 1.29
	Sen	97.12 ± 1.38	96.37 ± 1.15	96.38 ± 1.31
	Spc	96.28 ± 1.46	97.49 ± 1.63	96.49 ± 1.68
	Per	97.07 ± 1.37	97.69 ± 1.22	95.93 ± 1.58
Bayes	Acc	97.38 ± 1.31	97.85 ± 1.24	96.96 ± 1.79
	Sen	96.21 ± 1.18	96.93 ± 1.36	95.41 ± 1.43
	Spc	97.36 ± 1.23	97.27 ± 1.33	95.57 ± 1.52
	Per	96.87 ± 1.45	97.28 ± 1.47	96.32 ± 1.48
Ensemble	Acc	98.26 ± 1.27	97.95 ± 1.18	98.27 ± 1.37
	Sen	98.39 ± 1.04	98.01 ± 1.09	97.76 ± 1.46
	Spc	97.63 ± 0.95	97.98 ± 1.27	97.83 ± 1.45
	Per	98.84 ± 1.01	98.21 ± 0.98	98.46 ± 1.33

A closer look at the recent results in Tables 4–6 shows that classification performance while using the mixture model is relatively high and higher than several previous studies such as (Chen et al., 2014; Sayed et al., 2017). Referring to Table 5, we can even determine the type of artifact with an accuracy of more than 75% in all cases and datasets. It is noticeable in Table 6 that all components, regardless of the datasets, are classified into two classes with accuracy and sensitivity of more than 96% and 95%, respectively.

### 3.1 Simulated data results

**FIGURE 4:**
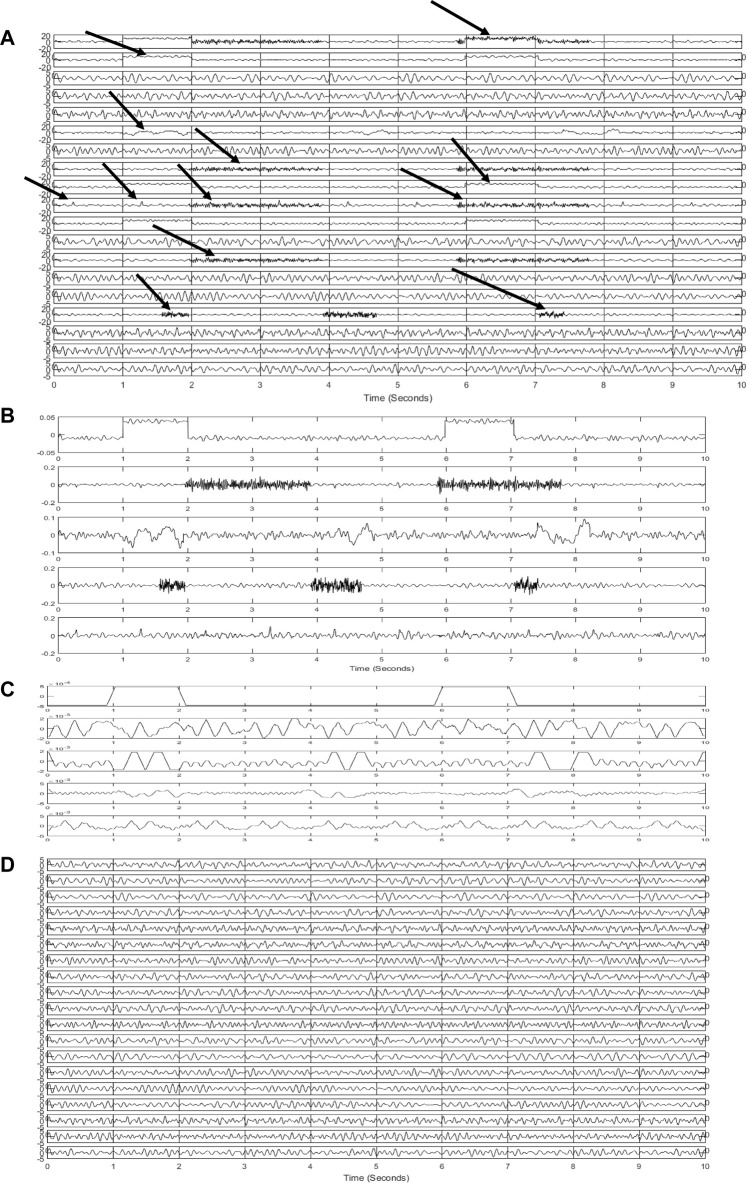
Results of the proposed method for simulated signals **(A)** simulated and contaminated EEG **(B)** detected artifactual components **(C)** denoised components using SWT **(D)** reconstructed EEG.

In Figure 4A, artificial and contaminated EEGs are represented. Artifacts can be seen in this figure. Some of them are pointed out by arrows. Simulated artifacts in this figure correspond to the mentioned artifacts in Figures 4A,B demonstrates extracted sources *via* the SOBI algorithm. First, three sources and the last one pertain to the artifacts. These sources are identified as artifacts using the ensemble of classifiers. EEG is leaked to these sources. Artifactual sources are taken into the SWT-based artifact removal algorithm in order to get artifacts eliminated. Figure 4C shows the output sources of SWT. Then, EEG channels are reconstructed using the inverse of the mixing matrix. Figure 4D represents the final reconstructed EEGs. Considering the pure EEGs and the results of the proposed method, no appreciable artifact is notified in the results. Moreover, reconstructed EEGs are justified by experts to evaluate the results visually.

**FIGURE 5:**
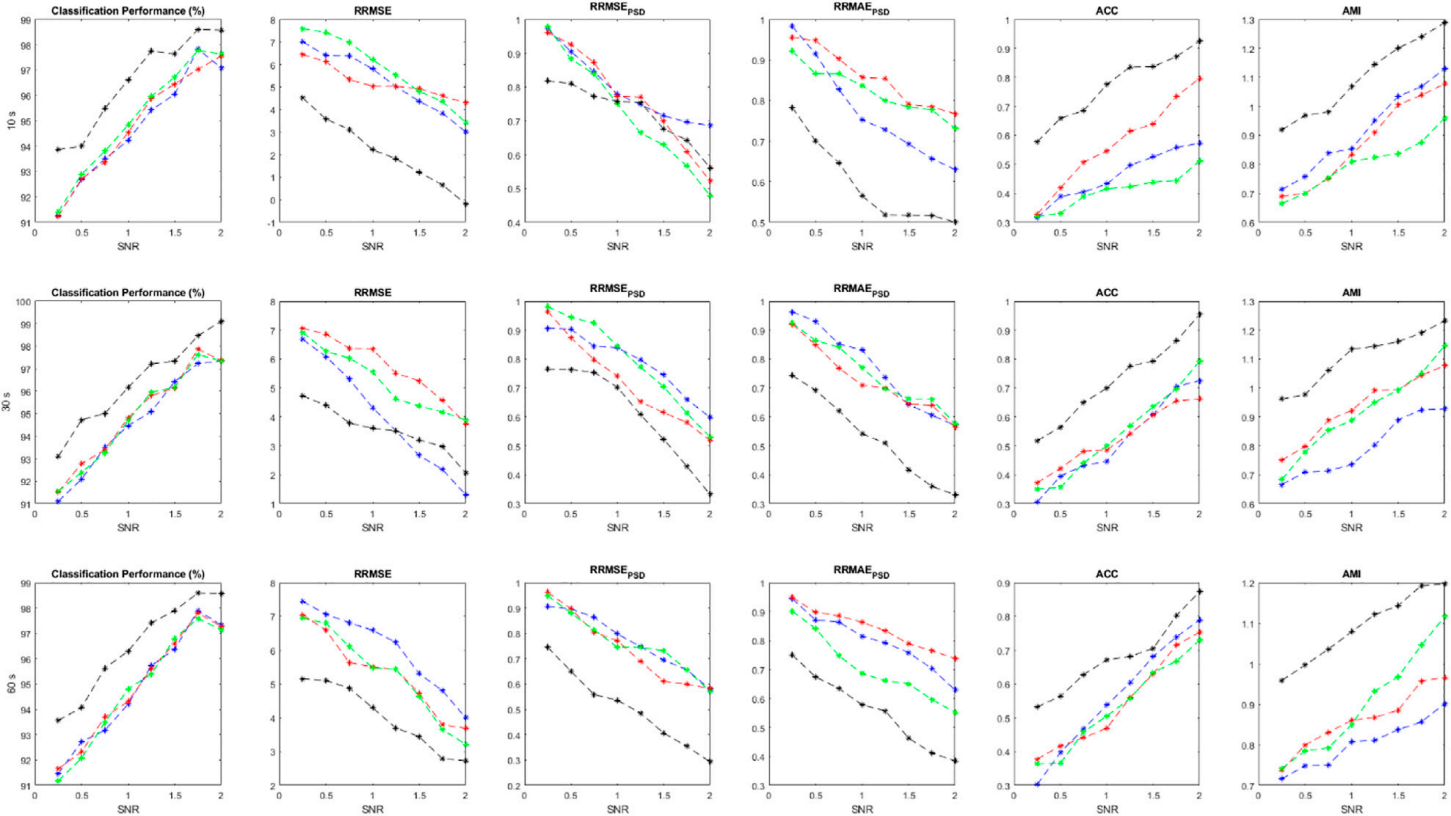
Performance parameters using simulated data at various SNR value and evaluation criteria, including ACP , RRMSE , $RRMSE_{PSD}\;$ , $\;RRMAE_{PSD}$ , ACC , and AMI .

In this section, we evaluate the proposed method through 200 independent realizations. In each realization, artifacts are separately generated at random intensities and then added to the simulated EEGs. The implementation is carried out at different SNR values to evaluate the method more precisely. ACP , RRMSE , $RRMSE_{PSD}\;$ , ACC , and AMI are calculated for each implementation and shown in Figure 5. We can compare the effectiveness of different classifiers trained with the suggested features. Average values for all performance measures are displayed for different length of signals and at different SNRs.

**FIGURE 6:**
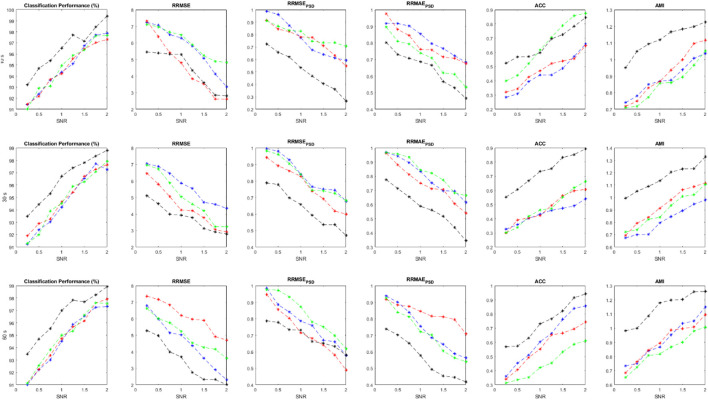
Performance measures for SSCEEG1 using evaluation criteria including ACP , RRMSE , $RRMSE_{PSD}\;$ , $\;RRMAE_{PSD}$ , ACC , and AMI .

**FIGURE 7:**
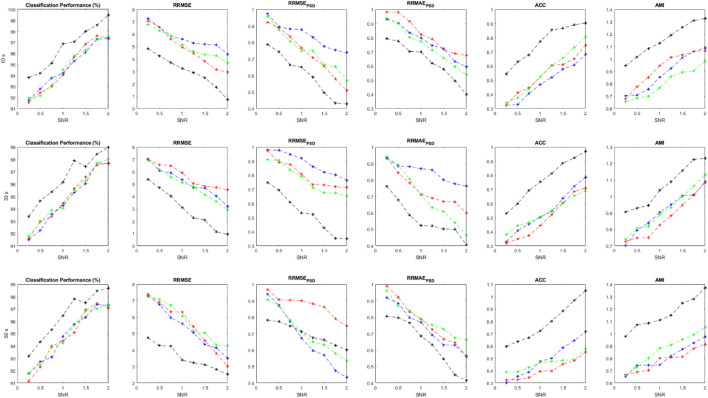
Performance measures for SSCEEG2 using evaluation criteria including ACP , RRMSE , $RRMSE_{PSD}\;$ , $\;RRMAE_{PSD}$ , ACC , and AMI .

As it is clear, the ensemble of classifiers outperforms the other classification models at all SNR values. All performance criteria are almost close for MLP, KNN and Bayes but the ensemble classification model is more effective. It is worth mentioning that the ensemble of classifiers can preserve the original EEGs when they are highly contaminated (e.g., SNR<0.5). Average values for each performance parameter at different SNRs are reported and displayed. As SNR decreases, all performance criteria degrade sharply. For Figures 5–7, Blue, red, green and black colors represent the results of bayes, KNN, MLP, and the ensemble classification model, respectively. The first, second, and third rows show the results for 10-s, 20-s and 30-s EEG signals, respectively. In each figure, the horizontal axis represents the signal-to-noise ratio (SNR) while the vertical axis shows the value for the evaluation criterion which was used.

The statistical analysis in Table 7 shows that the results of the ensemble model are significantly different from the sole classifiers. In all evaluation criteria, all *p*-values are below the confidence interval, suggesting that the proposed method can introduce a new algorithm that is much more effective than conventional classifiers. However, other classification models are not statistically different regarding the results and the employed statistical analysis. Considering AMI, RRMSE, and RRMSE_PSD_, it should be noted that these criteria are statistically different between the classifiers and also the ensemble model.

**TABLE 7:** Statistical analysis of the results in Figure 5 t-test analysis was conducted to check which criteria are significantly different. A confidence interval equal to 0.05 was considered in this analysis. *p*-values are reported in this table.

#		Classification accuracy	RRMSE	RRMSE_PSD_	RRMAE_PSD_	ACC	AMI
1	Bayes	0.0467	0.0491	0.0731	0.0436	0.0693	0.0481
2	KNN	0.0915	0.0458	0.0838	0.0484	0.0912	0.0498
3	MLP	0.0413	0.0786	0.0422	0.0321	0.0551	0.0401
4	Ensemble	0.0202	0.0374	0.0484	0.0396	0.0235	0.0218

### 3.2 Semi-simulation results

We collected two different datasets for semi-simulated signals to investigate the method’s performance more precisely. The proposed method is applied to both semi-simulated datasets. Figure 6 illustrates the results related to SSCEEG1. This dataset consists of actual pure EEGs contaminated by simulated artifacts. Similarly, Figure 6 indicates that the ensemble of classifiers leads to improved results for different signal lengths.

Since experts control the generated signals and label all of the extracted sources, we can easily measure the performance parameters. Additionally, we have the pure EEGs in both semi-simulated datasets. So evaluation measures containing ACP, RRMSE, RRMSE PSD, ACC, and AMI can be calculated for SSCEEG1 and SSCEEG2. Figure 7 shows the performance measures for the second dataset of semi-simulated EEGs called SSCEEG2 at different SNRs. Results for SSCEEG2 are quite similar to that of SSCEEG1. This similarity indicates that the considered EEG model to generate EEGs for the simulated dataset is quite reliable and realistic.

The results prove the proposed model for pure EEGs and also contaminated and simulated EEGS. Since the results for SSCEEG2 are almost close to that of SSCEEG1, we conclude that the proposed method is practical in real applications.

### 3.3 Real data results

**FIGURE 8:**
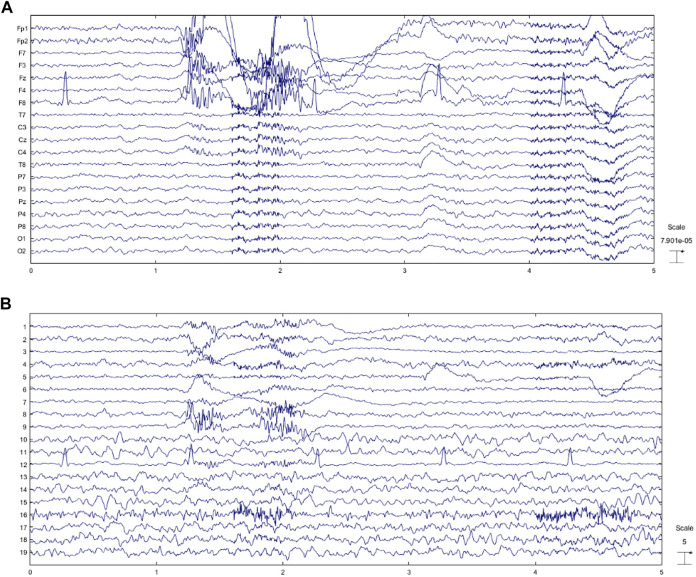
**(A)** Real contaminated EEG and **(B)** extracted sources using SOBI.

In this section, we apply the proposed method to real contaminated EEGs. These EEGs contain severe artifacts to evaluate the suggested artifact removal procedure. Since there is no ground truth available for real data, we cannot report performance parameters. In other words, for real EEGs evaluation procedure is performed quantitatively, including visualization criteria like topography or spectral density and temporal analysis. It should be noted that source classification accuracy for real data is previously reported in Table 3. For real contaminated EEGs visual inspection is performed by experts to evaluate the proposed method. Figure 8 shows a real contaminated EEG recording from a participant in a 5-s segment.

**FIGURE 9:**
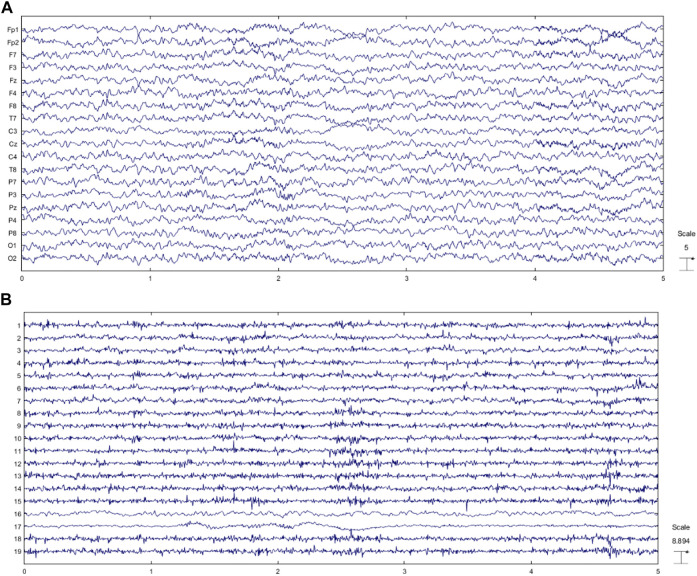
**(A)** Reconstructed EEG through the proposed method and **(B)** EEG components.

As can be seen, SOBI separates sources and isolate artifacts. Although the source separation algorithm is effective, brain activity leaks to most artifactual sources. This motivates us to employ automated artifact detection using the ensemble of mentioned classifiers. All detected artifact components are processed *via* the proposed artifact elimination method based on SWT. In Figure 8, an EEG recording contaminated with all mentioned artifacts is represented. Taking a closer look at extracted sources in Figure 8B, it is clear that sources 1,2,3,4,5,6,7,8,9,12, and 16 are artifactual. All of these sources are detected by the proposed classification model. Figure 9 shows the reconstructed EEG and its sources after artifact removal.

**FIGURE 10:**
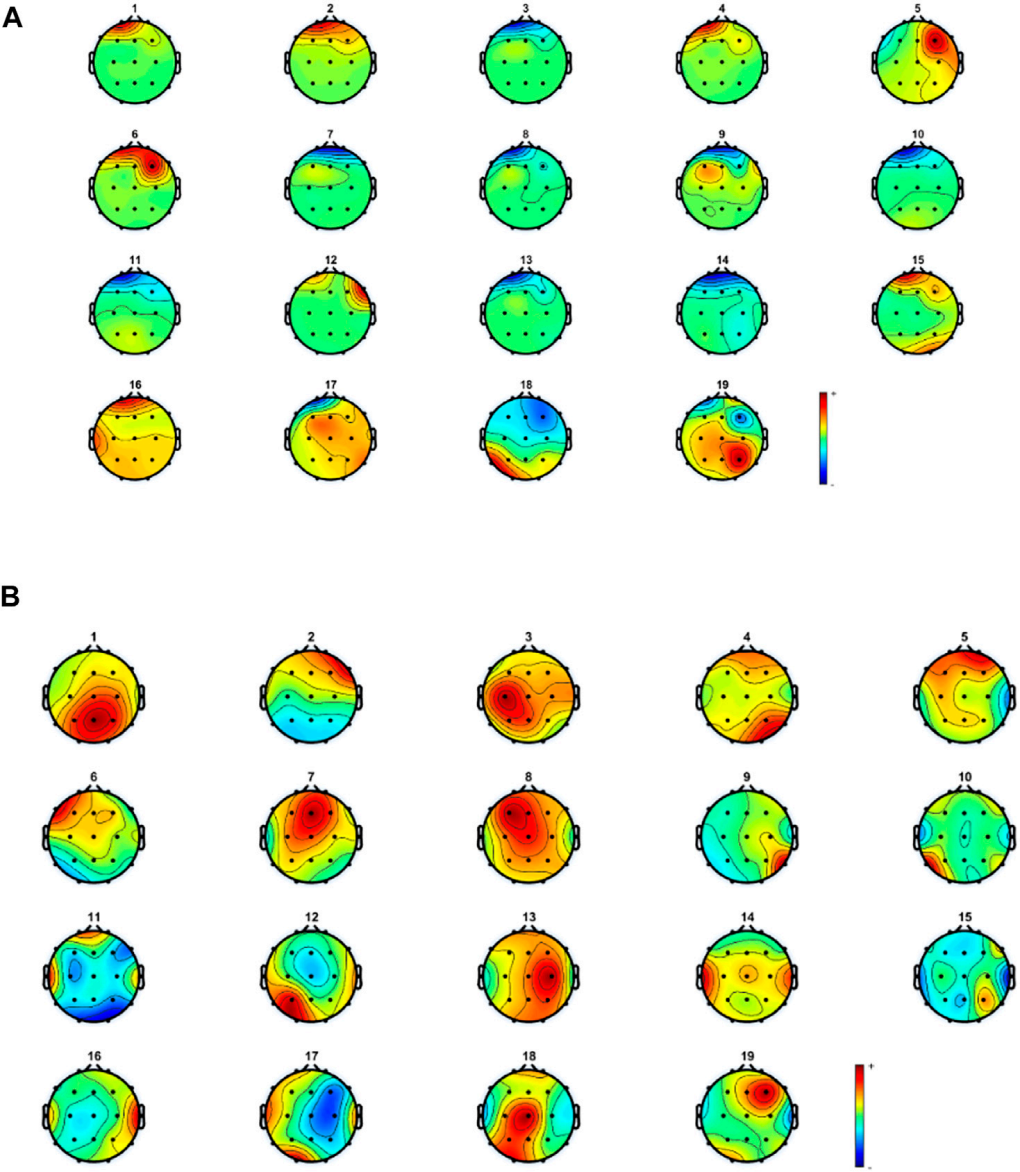
Topography maps for components related to **(A)** contaminated and **(B)** reconstructed and cleaned EEG.

**FIGURE 11:**
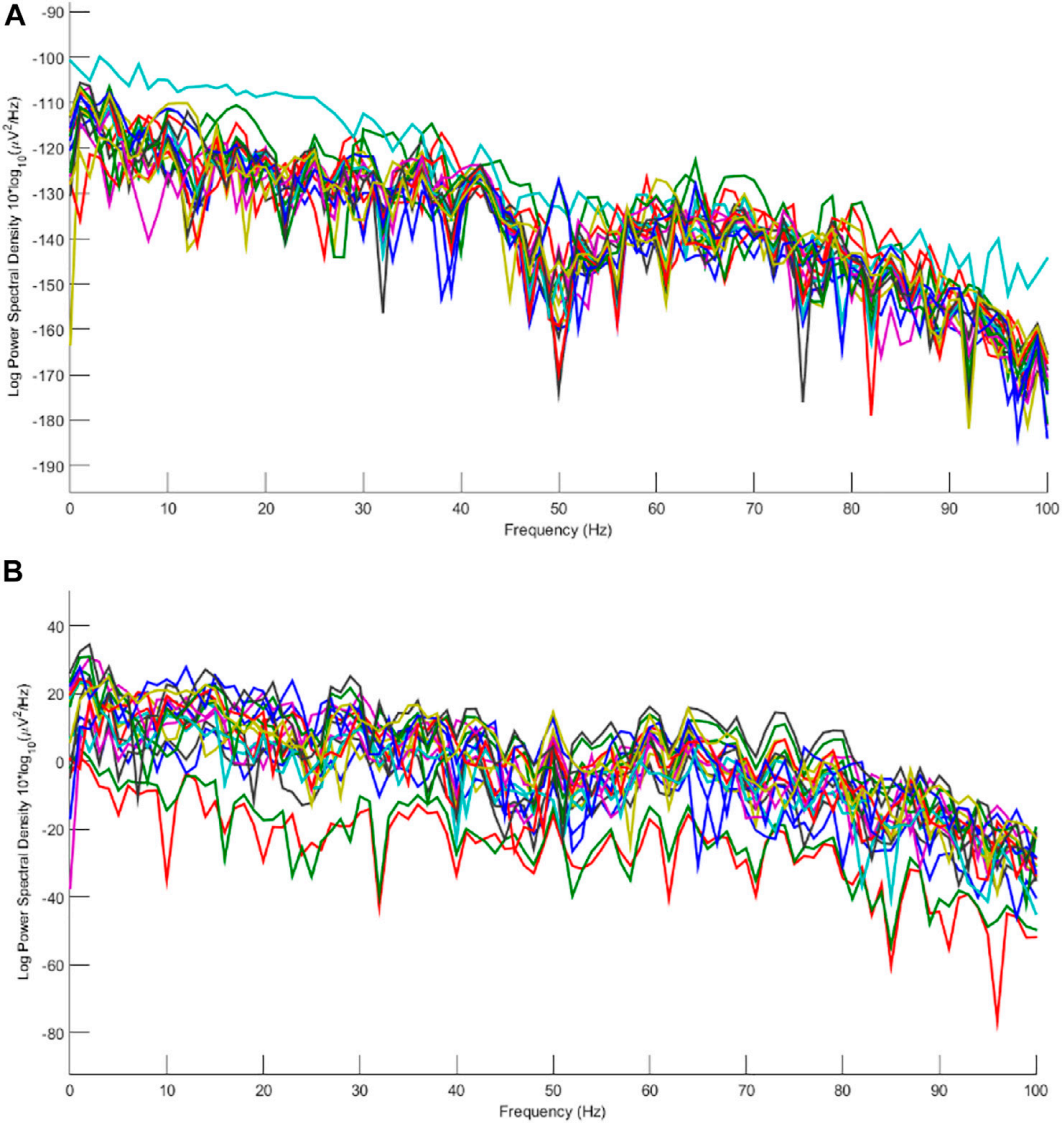
Power spectral density of channels for **(A)** contaminated real and **(B)** cleaned EEGs.

Some muscle activity can be seen in Fp1, Fp2, and F8. Moreover, it might be realized that artifacts related to eye movement and blinking still remain in the reconstructed EEG. To analyze the results more thoroughly, we decided to consider the topography maps and power spectral density before and after applying the proposed method. Figure 10 illustrates the topography maps for extracted sources; similarly, Figure 11 represents power spectral density for channels.

**FIGURE 12:**
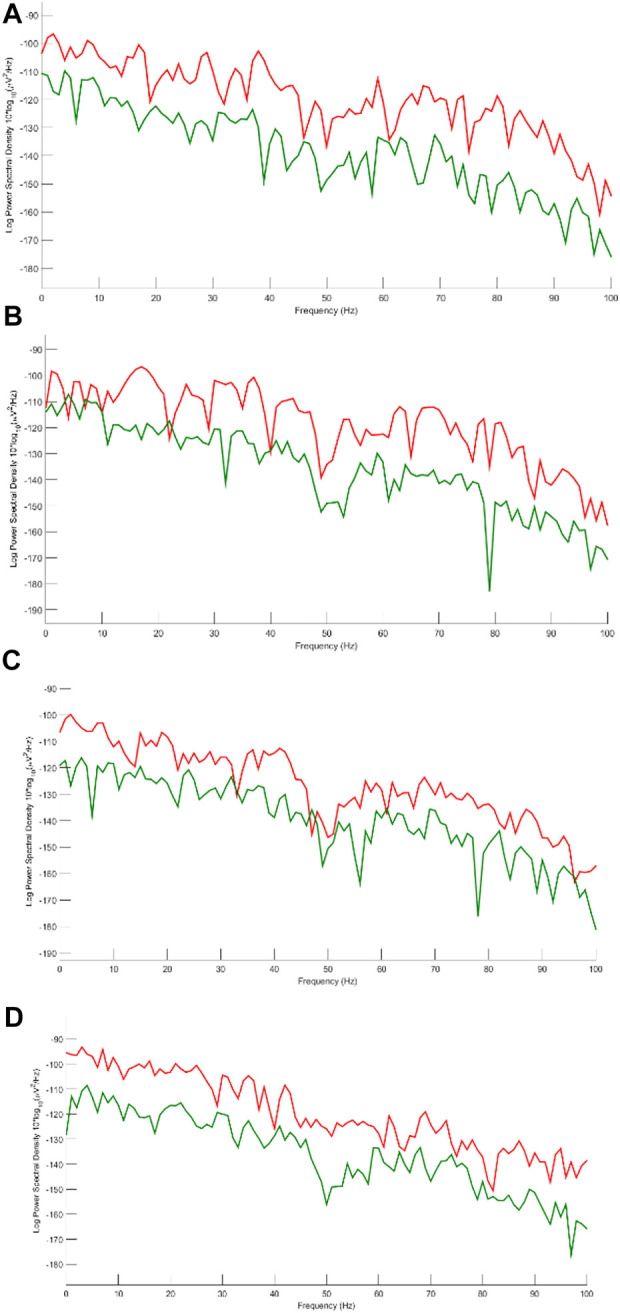
Power spectrum for **(A)** Fp1, **(B)** Fp2, **(C)** F8 and **(D)** T7. Red and green lines correspond to EEGs before and after applying artifact elimination respectively.

Considering the results in Figure 11, artifacts are easy to distinguish in most components. Eye movement and blinking are clear with respect to the channel locations. The 12th component, for example, shows the activity in both sides of the forehead, which is related to ECG and can be seen in F8 channel. The topography map for components after artifact removal ensures the proposed method works effectively. Figure 12 shows the power spectral density for all channels before and after the proposed method.

We decided to study the results in the frequency domain more comprehensively. Therefore, four channels including Fp1, Fp2, F8, and T7 contaminated by severe artifacts are selected, and the power spectral density for those channels are represented in Figure 12.

All of these channels are detected as artifacts by the classification model. Since brain activity mostly concentrates within the frequency band 2–30 Hz and considering that all participants are in the rest mode sitting comfortably, we can easily analyze the power spectral density for given channels. Moreover, based on the previous studies (Lawhern et al., 2012; Chen et al., 2017; Yang et al., 2018) we can assume that most artifacts are in the frequency band 1–4 Hz and above 30 Hz. Considering this fact, we can realize that the proposed method can perform well in almost all types of artifacts. Channels Fp1 and Fp2 are severely interrupted by eye blinking and movement. Figure 10A shows that the SWT-based method can eliminate ocular activity. In addition, in these channels, there are some activities above 30 Hz, which is well suppressed by the suggested method. F8 and T7 are corrupted by ECG and muscle activity. Considering the spectrum of original and reconstructed signals around 40, 70, and 80 Hz, it is obvious that eliminating these artifacts is possible *via* the proposed method. The channel T7 contains noise and EMG, more or less. EEG content is preserved in almost all channels with a little desired information loss.

As stated in Eq. 8, PSD_tr_ was introduced as a reliable criterion in (Fang et al., 2015) to evaluate artifact removal methods. We also considered this measure and calculated in our implementations. Table 8 represents $PSD_{tr}$ for all datasets used in this study.

**TABLE 8:** $PSD_{tr}$ for simulated, semi-simulated and real EEGs at signal length 10, 30, and 60 s using four proposed classifiers.

Length (s)	Classifier	SCEEG	SSCEEG1	SSCEEG2	RCEEG
10	MLP	0.36±0.12	0.44±0.13	0.46±0.14	0.57±0.13
	KNN (K = 20)	0.41±0.11	0.43±0.16	0.44±0.16	0.60±0.12
	Bayes	0.38±0.09	0.48±0.19	0.49±0.16	0.61±0.14
	Ensemble	0.37±0.08	0.40±0.13	0.44±0.12	0.58±0.09
30	MLP	0.39±0.15	0.51±0.14	0.53±0.17	0.53±0.16
	KNN (K = 20)	0.42±0.16	0.47±0.13	0.45±0.18	0.57±0.19
	Bayes	0.43±0.16	0.53±0.16	0.57±0.21	0.68±0.22
	Ensemble	0.32±0.14	0.45±0.14	0.49±0.17	0.71±0.17
60	MLP	0.48±0.18	0.53±0.15	0.52±0.16	0.58±0.19
	KNN (K = 20)	0.50±0.14	0.52±0.17	0.53±0.19	0.61±0.21
	Bayes	0.46±0.13	0.49±0.16	0.51±0.18	0.53±0.23
	Ensemble	0.45±0.10	0.48±0.13	0.50±0.17	0.52±0.16

The average value of this measure is computed over all contaminated EEGs. The average value of the aforementioned performance measure is 0.2496 (for the proposed method) over all EEG simulations and recordings. This measure is calculated in order to compare this study with previous ones. Results show that the proposed method outperforms most previous studies in this field.

## 4 Discussion

In this study, we managed to suppress different artifacts through an automated procedure. To investigate more, we decided to examine the proposed angle plot in representing signals. Supplementary Figure S1 shows the angle plot for some well-known signals whose dynamics are clear to us. As illustrated, angle plot can accurately describe signals’ dynamics appropriately. The sinusoid signal has the frequency equal to 10 Hz. The random time series is zero-mean with unit variance. The chaotic signal is achieved by employing the logistic regression with the tuning parameter equal to 3.9. We decided to reconstruct the angle plot for all types of artifactual components. Expectedly, angle plots are significantly different and can be recognized visually. These simulations showed that the proposed method could effectively reflect the dynamics of simple and complex signals alike.

In contrast to several studies such as (Delorme et al., 2007; Hoffmann and Falkenstein, 2008; Chen et al., 2017), the proposed method in this study is completely automated and more precise. Since previous studies use different performance criteria, it is impossible to compare their results with ours. But we have tried to compute major evaluation parameters to have a fair comparison. In addition, previous studies have applied their methods to different datasets. So it seems to be difficult to compare the results. In terms of computation complexity, it takes the proposed method less than 0.25 s to analyze a 1-min and 19-channel EEG recording sampled at 256 Hz and remove artifacts. For all simulations and recordings, the processing time is under 0.25 s, which is practical for BCI applications and also for diagnosis purposes. All implementations are performed using MATLAB (release R2016a) running on Windows 7 Laptop PC with Intel (R) Core (TM) 2 Duo 2.0 GHz processor with 4 GB RAM. The average processing time and the standard deviation for real EEG recordings and simulated EEGs are 0.21 and 0.09 s, respectively. Since other similar methods are compared with SWT-based artifact removal in (Hoffmann and Falkenstein, 2008), we avoid reviewing them here for the sake of space. In this study, since only artifactual components are fed into the SWT-based artifact removal procedure, it is clear that the proposed method has less computation complexity than methods that analyze all components.

Supplementary Table S1 shows the average processing time and the standard deviation for all components at the signal lengths of 10, 30, and 60 s. Other BSS methods might be suggested in some studies like (Chen et al., 2017), which claim that some other BSS methods outperform SOBI in particular situations, but while considering all evaluation measures such as processing time and simplicity, it is evident that SOBI is slightly better than most BSS methods.

Since support vector machine (SVM) is one of the most effective classifiers in previous studies like (Brychta et al., 2007a; Delorme and Makeig, 2004), we decided to employ SVM with the polynomial kernel (order of 3). The SVM’s kernel and also other parameters are set based on trial and error. SVM Classification results are no better than other mentioned classifiers. The reason is mainly related to the separability in the feature space. Ten-fold cross-validation is performed to evaluate SVM. Results show that the mixture classification model can slightly better recognize components. One can say that SVM can also be included in the mixture. This motivates us to build the mixture classification model using SVM. Table 9 represents the classification results. Components are classified into “brain-activity” and “artifacts” groups.

**TABLE 9:** Classification accuracy of all the classifiers and also the ensemble of them. Average values are mentioned here.

Classifier		SCEEG	SSCEEG1	SSCEEG2	RCEEG
SVM	Acc	96.76±1.13	96.63±1.26	95.26±1.23	95.66±1.14
	Sen	96.34±1.09	96.17±1.15	96.39±1.16	96.73±1.17
	Spc	96.91±1.17	95.24±1.01	94.29±1.39	96.54±1.21
	Per	97.07±0.96	96.39±1.12	95.09±1.08	96.21±1.18
MLP + KNN + Bayes + SVM	Acc	99.39±0.75	99.28±0.64	98.22±0.91	98.78±1.01
	Sen	99.12±0.82	98.92±0.57	98.84±0.77	98.39±0.94
	Spc	98.86±0.67	98.76±0.99	99.03±0.82	99.08±0.85
	Per	99.26±0.52	98.51±0.97	98.47±0.88	98.94±0.97

For the sake of space, we decided to bring average values for 10, 30 s, and 1-min components in each dataset. Results in Table 9 suggest that the proposed mixture is able to recognize artifacts and separate them from brain activity. The final mixture of classifiers, including SVM, is more accurate than other proposed classification models. So we can realize that this mixture can be performed in the future. Additionally, SVM’s results are slightly better than MLP and Bayes, but statistical analysis shows no difference between classifiers while used alone. In contrast to single classifiers, ensemble classification models have better results and higher accuracies. Based on the t-test, both mixtures (MLP + KNN + Bayes and MLP + KNN + Bayes + SVM) have significantly higher results than other classification models. As it is mentioned, we apply the voting. In terms of the 3-classifier mixture, one class easily has more votes, but in the 4-classifier mixture, if the votes are equal, we go for SVM’s vote since it has slightly better results than other classifiers.

As was mentioned in the results, we have two scenarios, including 6-class and binary classification. The former is about recognizing the artifact type, such as EOG, ECG, etc., while the latter is focused on just recognizing artifactual and “clean” EEG components. Looking back at the classification results in Tables 3–5, we conclude that the binary classification had higher recognition rates compared to the 6-class scenario. The reason could be the similarity that some artifacts have in the proposed features. Our suggested attributes can definitely distinguish artifacts from neural components, however, they are not as effective when it comes to artifact type recognition. In addition, some unpredictable errors might have happened while labeling artifactual components as there is always information leakage between components while employing BSS methods. It means that estimated artifactual components do not entirely belong to just one specific artifact type; rather, they carry information about two or more artifacts in one EEG component. Moreover, experts might have made mistakes while recognizing and labeling EEG components. The chosen features are the ones which have worked successfully in EEG dynamics representation in our previous studies. That could be the main reason why these features work well in EEG neural and artifactual component recognition. These features were selected after we tested several features from time, frequency, and time-frequency analysis in previous studies. Angle plot has the potential to be described in several ways, such as in the framework of graph theory or as a complex network which makes this processing method appropriate in EEG analysis. As reported above, the ensemble of classifiers shows higher classification accuracies compared to the sole classifiers. As mentioned, according to the statistical analysis, there is not a significant difference between the results reported by t-test analysis. This suggests that maybe in future studies, other fusion methods such as bagging and boosting methods could be employed to achieve better and significantly different results of using fusion methods. The other aspect could be the proposed features which have resulted in similar results for all the classifiers. It could be inferred that in terms of the misclassified samples, all classifiers mistakenly labeled specific samples. However, after error analysis, it turned out that this hypothesis was correct and some samples which were not outliers were misclassified by all of our classifiers. On the other hand, the proposed fusion method could not make a considerable difference as for those samples, all classifiers mislabeled data. It could be suggested that, in future studies, other features from the EEG angle plot could be extracted and analyzed. It is worth mentioning that our suggested approach resulted in almost 98% accuracy (on average), according to Table 9. In comparison with previous studies in this area, this is an acceptable classification performance considering that we have analyzed several scenarios and different datasets, such as real EEG signals, and simulated and semi-simulated ones.

Taking a closer look at Figures 5–7, we can conclude that the evaluation criteria, including ACP , RRMSE , $RRMSE_{PSD}\;$ , $\;RRMAE_{PSD}$ , ACC , and AMI are proportionate with SNR. Although there is not a considerable gap in classification accuracy between the sole classifiers and the ensemble of them, in other criteria such as RRMSE in both time and frequency domains, we can see that there is a considerable difference between the ensemble model and sole classifiers. However, for long-windowed 60-s EEG components, the ensemble model does not work well. It could be related to the non-stationary nature of EEG signals as EEG dynamics, characteristics, and statistical features change in long windows of signals. On the other hand, RRMSE in the frequency domain shows that the ensemble model has better performance than the classifiers. For RRMSE in the frequency domain, the power spectrum density of signal should be estimated, which is a serious problem in spectrum estimation as it is highly dependent on the signal length, number of segments, and the overlap used to estimate the EEG spectrum density. This could affect our estimation and lead to different results. This could justify the difference in the trends of RRMSE in time and frequency domains. Although they follow the same pattern in lower SNRs, in higher SNRs they follow different trends. Other evaluation criteria, including ACC and AMI are used to measure similarity between the clean EEG signal and the reconstructed one. However, due to the nonlinearity in EEG dynamics, we believe AMI can represent this similarity better as it works for nonlinear signals better than ACC, which is more suitable for determined signals. Both AMI and ACC show that there is a considerable difference in the performance of the ensemble model and sole classifiers. This again implies that although the suggested approach does not stand out in terms of classification performance, but considering the whole process, our methods is effective in EEG artifact detection and elimination.

We prefered to analyze the feature space more. To do that, all components from all datasets are normalized and then 12 suggested features are extracted from each source. We perform principal component analysis over all samples from different datasets and then normalize the components to achieve main components from the feature space. The two first components are plotted in Supplementary Figure S2. Red and blue circles indicate artifactual and neural components respectively. 2,000 samples are selected randomly from each class i.e. neural activity and artifacts to have equal number of samples in each class.

It can be verified that the proposed features efficiently determine artifactual and neural components in this study. Results show that although classification performance is the same for almost all components at different length, 10-s components are better classified in comparison with components with the length of 30 and 60 s. As it is mentioned before, the phase space and consequently the angle space are able to demonstrate and represent the signal dynamics even at short length of the signal. This nonlinear analysis provides us with some new features which do not vary based on the length of the signal. That is why we can recognize sources very well regardless to the signal length. We have also tried different sampling rates. Real and simulated signals are sampled at the sampling rate of 128, 256, 512, and 1,024 Hz. No significance difference is found in the results between different sampling rates. Similar results at different signal length and sampling rates suggest that the proposed method is practical for different purposes. Also, all results are acceptable and comparable in terms of visual subjective inspection and also quantitative objective measures. In this study, we focus on removing stereotyped biological artifacts. Non-stereotyped artifacts such as head and electrode movement might cause special patterns while recording EEG. These artifacts should be eliminated before the proposed artifact removal procedure. Fortunately, these artifacts can be easily discarded from the data by visual inspection. In term of computer simulations, all simulations have been implemented in MATLAB. We also used of EEGLAB (Delorme and Makeig, 2004).

EEG signal, as mentioned before, is a complex, chaotic, nonlinear and dynamic biosignal whose characteristics are nonstationary. This means that proposed EEG preprocessing methods should preserve the main nonlinear characteristics of preprocessed EEG signals. Deep learning models are one of those methods which can provide this feature, and thanks to the advancement in data recording systems and data repositories, now it is possible to employ deep learning in most projects. Deep learning models can learn high-level and hierarchical data representations from big and massive data, which is why deep learning has been widely used in signal processing, specifically in EEG artifact removal. Several studies, such as (Yang et al., 2018; Sun et al., 2020; Joseph et al., 2021; Mathe et al., 2021; Webb et al., 2021; Zhang et al., 2021) have employed deep neural networks to detect and remove artifacts from EEG and other biosignals. The main disadvantage of deep learning-based methods is the number of samples they need to train their models, which makes these methods impractical in real-world applications as we have limited numbers of samples or subjects. In (Sun et al., 2020), it is shown that a one-dimensional residual convolutional neural network model (1D-ResCNN) can effectively suppress the EEG artifacts with much lower RMSE compared to ICA-based and also wavelet-based methods. However, in that study, the combination of SOBI and wavelet or the combination of ICA and wavelet denoising was not studied, and also just ECG, EMG, and EOG artifacts were considered and analyzed in their implementation. Despite interesting results, in that study, just SNR and RMSE were reported, which made us unable to compare all the before-mentioned criteria we used to evaluate our methods. As it is highly recommended, in noise reduction algorithms, one should consider temporal, and frequency domains of preprocessed EEG signals as well as topography maps to evaluate the artifact removal method fairly. Other studies such as (Mathe et al., 2021) also reported outstanding results which were not compared to the conventional methods in this field. These studies also did not consider all scenarios such as simulated, semi-simulated, and real EEG signals to test their proposed methods. To sum up, deep learning-based EEG noise/artifact removal methods have reached considerable and outstanding results. However, more analyses are required to study deep neural networks in this area completely. As it is stated in those articles, their proposed methods need a better design for practical and real-world applications. Also, all types of artifacts should be considered to evaluate how effective such methods are in EEG artifact removal (Mathe et al., 2021). More future studies should be conducted to compare the methods which are more based on signal processing basis with the ones employing deep learning. We can now implement most complex deep learning neural networks thanks to computer hardware advancements.

On the other hand, our proposed method can be considered a novel method in signal processing or time series analysis. Several methods in complex time series analysis called complex networks have recently gained attention from different fields of science. These complex networks which are reconstructed from nonlinear time series, can represent them in a new space using graph theories. In other words, such graphs are driven from complex time series and provide new representations. Several complex networks such as visibility networks (graphs) have been introduced do far (Mohammadpoory et al., 2017; Zou et al., 2019). Apparently, our proposed angle plot can be viewed as a visibility graph; however, it is reconstructed based on the angle values in the state space. In other words, our angle network could be described as an unweighted visibility network driven from angle values in EEG state space. More exploration could be performed in future studies to compare our proposed angle network with other successful complex networks.

## 5 Conclusion

In this paper, we introduce a new method to suppress different types of artifacts and noise based on BSS (SOBI), wavelet transform (SWT) and an ensemble of classifiers (MLP, KNN, Bayes, and SVM). A preprocessing chain is suggested and evaluated in this paper. We have concluded that the proposed method is effective, fast and simple. Based on the results, hybrid methods, including BSS methods and artifact elimination procedures, are recommended to remove artifacts and noise from EEG (Yang et al., 2018). Automated methods are superior to methods based on visual inspection in terms of artifact elimination and EEG interpretation (Romo Vázquez et al., 2012; Cao et al., 2015; Islam et al., 2016; Yang et al., 2018). We proposed an automated EEG artifact removal approach using SOBI, conventional classifieirs and SWT to reduce stereotyped EEG biological artifacts.

The proposed method has some advantages, such as its simplicity which makes it reproducible in real-world applications. The suggested approach can be used in online or real-time EEG pre-processing platforms. The most challenging problem with our proposed method is that this approach is computationally extensive, and fast processing machines are required to implement this method. Since the ensemble of several classifiers is employed in this study, it should be noted that over-fitting and under-fitting might cause some problems. It can be considered one of the weak points while applying classifiers in automated artifact recognition methods. This problem could be tackled by considering training and testing errors together and also performing some validation methods such as k-fold cross-validation. In addition, the proposed artifact detection method is based on SOBI, which is mainly effective for stereo-typed artifacts like the ones we had in most research experiments, while in other practical ones, such as newborn’s EEG preprocessing, we mostly face non-stereotyped artifacts (Kumaravel et al., 2022). This point should also be taken into consideration while employing our proposed methods. Another important point is about low-dimension EEG signals. This study assumes that the number of sources is equal to or less than the number of channels. Therefore, sufficient EEG channels are required to estimate sources correctly. Moreover, it is considered that the number of artifacts is less than or equal to the number of components and channels. These assumptions might cause problems while dealing with low-dimension EEG signals. In that situation, some decomposition methods, such as empirical mode decomposition, might be a good solution to decompose EEGs as the first step. Then BSS methods can be applied to decomposed signals. Previous studies have shown that for EEG artifact removal, the combination of EEG subspace decomposition methods such as ICA-family methods and wavelet transforms could lead to acceptable results (Zikov et al., 2002; Brychta et al., 2007b; Delorme et al., 2007; Kumaravel et al., 2022). We used this to propose our method, which is mainly based on SOBI and SWT. We chose SOBI for EEG subspace decomposition and SWT for the wavelet transformation due to their high performance in the previous studies. These methods, as stated above, have their own shortcoming and are not necessarily “the best” or “the superior” methods in EEG artifact removal. It should be noted that other methods should also be considered and could be compared with ours in future studies to explore more in this field.

No global measure is available to compare different methods in this field. Besides, previous studies have tested methods on different datasets. This makes the results inconsistent. That is why most previous studies have trouble reproducing other methods. We have tried to evaluate the proposed method through several approaches. Temporal and spectral criteria are considered. We will try to define new evaluation criteria in future work. It should be noted that the results suggest that although the proposed method outperforms most previous studies and is fast, effective and practical, it fails in a few cases while dealing with highly-contaminated EEGs. The proposed method has been applied to real, semi-simulated, and simulated EEGs. In our future studies, we are going to compare different methods with the present one. In addition, the proposed method could be employed to eliminate other types of artifacts, such as power-line interference and head movement.

## Data Availability

The original contributions presented in the study are included in the article/Supplementary Material, further inquiries can be directed to the corresponding author.

## Supplementary material

SUPPLEMENTARY FIGURE S1 Scatter plot of the two largest principal components of the feature space over all datasets.

SUPPLEMENTARY FIGURE S2 Angle plots for single-channel artifactual EEGs severely contaminated by **(A)** ECG, **(B)** EMG, **(C)** EOG, **(D)** eye blinking and **(E)** white noise. **(F)** Angle plot for artifact-free EEG. All signals are sampled at 256 Hz and have a length equal to 10 s.

## References

[B1] Acharya U. R. Molinari F. Sree S. V. Chattopadhyay S. Ng K. H. Suri J. S. (2012). Automated diagnosis of epileptic EEG using entropies. Biomed. Signal Process. Control 7, 401–408. 10.1016/j.bspc.2011.07.007

[B2] Bai Y. Wan X. Zeng K. Ni Y. Qiu L. Li X. (2016). Reduction hybrid artifacts of EMG-EOG in electroencephalography evoked by prefrontal transcranial magnetic stimulation. J. Neural Eng. 13, 066016. 10.1088/1741-2560/13/6/066016 27788128

[B3] Belouchrani A. Abed-Meraim K. Cardoso J. F. Moulines E. (1993). Second-order blind separation of correlated sources. Proc. Int. Couf. Digital Sig. Proc., Cyprus, 346–351.

[B4] Belouchrani A. Abed-Meraim K. C Cardoso J. Moulines E. Nicole R. (1997). A blind source separation technique using second-order statistics. IEEE Trans. Signal Process. 45, 434–444. 10.1109/78.554307

[B5] Brychta R. Shiavi R. Robertson D. Diedrich A. (2007a). Spike detection in human muscle sympathetic nerve activity using the kurtosis of stationary wavelet transform coefficients. J. Neurosci. Methods 160 (2), 359–367. 10.1016/j.jneumeth.2006.09.020 17083982 PMC2075105

[B6] Brychta R. Tuntrakool S. Appalsamy M. Keller N. Robertson D. Shiavi R. (2007b). Wavelet methods for spike detection in mouse renal sympathetic nerve activity. IEEE Trans. Biomed. Eng. 54, 82–93. 10.1109/TBME.2006.883830 17260859 PMC2075098

[B7] Bui T. D. Chen G. (1998). Translation-invariant denoising using multi-wavelets. IEEE Trans. Signal Process. 46 (12), 3414–3420. 10.1109/78.735315

[B8] Cao K. Guo Y. Su S. W. (2015). “A review of motion related EEG artifact removal techniques,” in 2015 9th International Conference on Sensing Technology (ICST), Auckland, New Zealand, 08-10 December 2015, 600–604. 10.1109/ICSensT.2015.7438469

[B9] Cardoso J.-F. Souloumiac A. (1996). Jacobi angles for simultaneous diagonalization. SIAM J. Matrix Anal. Appl. 17 (1), 161–164. 10.1137/s0895479893259546

[B10] Castellanos N. P. Makarov V. A. (2006). Recovering EEG brain signals:artifact suppression with wavelet enhanced independentcomponent analysis. J. Neurosci. Methods 158, 300–312. 10.1016/j.jneumeth.2006.05.033 16828877

[B11] Chen M. Fang Y. Zheng X. (2014). Phase space reconstruction for improving the classification of single trial EEG. Biomed. Signal Process. Control 11, 10–16. 10.1016/j.bspc.2014.02.002

[B12] Chen X. Liu A. Chen Q. Liu Y. Zou L. McKeown M. J. (2017). Simultaneous ocular and muscle artifact removal from EEG data by exploiting diverse statistics. Comput. Biol. Med. 88, 1–10. 10.1016/j.compbiomed.2017.06.013 28658649

[B13] Chen X. Liu A. Chiang J. Wang Z. J. McKeown M. J. Ward R. K. (2016). Removing muscle artifacts from EEG data: Multichannel or single-channel techniques? IEEE Sens. J. 16, 1986–1997. 10.1109/jsen.2015.2506982

[B14] Coifman R. R. Donoho D. L. (1994). Wavelets and statistics, 103. NewYork: Springer-Verlag, 125–150. Springer Lecture Notes in Statistics.Translationinvariantde-noising

[B15] Croft R. J. Barry R. J. (2000). Removal of ocular artifact from the EEG: A review. Clin 30, 5–19. 10.1016/S0987-7053(00)00055-1 10740792

[B16] Delorme A. Makeig S. (2004). Eeglab: An open source toolbox for analysis of single-trial EEG dynamics including independent component analysis. J. Neurosci. Methods 134, 9–21. 10.1016/j.jneumeth.2003.10.009 15102499

[B17] Delorme A. Sejnowski T. Makeig S. (2007). Enhanced detection of artifacts in EEG data using higher-order statistics and independent component analysis. NeuroImage 34, 1443–1449. 10.1016/j.neuroimage.2006.11.004 17188898 PMC2895624

[B18] Fang Y. Chen M. Zheng X. (2015). Extracting features from phase space of EEG signals in brain–computer interfaces. Neurocomputing 151 (3), 1477–1485. 10.1016/j.neucom.2014.10.038

[B19] Goshvarpour Atefeh Abbasi Ataollah Goshvarpour Ateke (2016). Dynamical analysisof emotional states from electroencephalogram signals. Biomed. Eng. Appl. Basis Commun. 28 (2), 1650015. 10.4015/s1016237216500150

[B20] Hamaneh M. B. Chitravas N. Kaiboriboon K. Lhatoo S. D. Loparo K. A. (2014). Automated removal of EKG artifact from EEG data using independent component analysis and continuous wavelet transformation. IEEE Trans. Biomed. Eng. 61 (6), 1634–1641. 10.1109/TBME.2013.2295173 24845273

[B21] Hayashi K. Mukai N. Sawa T. (2015). Poincaré analysis of the electroencephalogram during sevoflurane anesthesia. Clin. Neurophysiol. 126 (2), 404–411. 10.1016/j.clinph.2014.04.019 24969375

[B22] Hoffmann S. Falkenstein M. (2008). The correction of eye blink artefacts in the EEG: A comparison of two prominent methods. PLoS ONE 3 (8), e3004. 10.1371/journal.pone.0003004 18714341 PMC2500159

[B23] Islam Md Kafiul Rastegarnia Amir Yang Zhi (2016). Methods for artifact detection and removal from scalp EEG: A review. Neurophysiol. Clinique/Clinical Neurophysiol. 46 (4–5), 287–305. 10.1016/j.neucli.2016.07.002 27751622

[B24] Joseph A. Makhoul A. Couturier R. Demerjian J. (2021). Deep recurrent neural network-based autoencoder for photoplethysmogram artifacts filtering. Comput. Electr. Eng. 92, 107065. 10.1016/j.compeleceng.2021.107065

[B25] Jug T. P. Makeig S. McKeown M. J. Bell A. J. Lee T. W. Sejnowski T. J. (2021). Imaging brain dynamics using independent component analysis. Proc. IEEE Inst. Electr. Electron Eng. 89, 1107–1122. 10.1109/5.939827 PMC2932458 20824156

[B26] Jung T. P. Makeig S. Humphries C. Lee T. W. McKeown M. J. Iragui V. (2000). Removing electroencephalographic artifacts by blind source separation. Psychophysiology 37, 163–178. 10.1111/1469-8986.3720163 10731767

[B27] Klemm M. Haueisen J. Ivanova G. (2009). Independent component analysis: Comparison of algorithms for the investigation of surface electrical brain activity. Med. Biol. Eng. Comput. 47 (4), 413–423. Epub 2009 Feb 13. PMID: 19214614. 10.1007/s11517-009-0452-1 19214614

[B28] Kumaravel V. P. Farella E. Parise E. Buiatti M. (2022). Near: An artifact removal pipeline for human newborn EEG data. Dev. Cogn. Neurosci. 54, 101068. 10.1016/j.dcn.2022.101068 35085870 PMC8800139

[B29] Lagerlund T. D. Sharbrough F. W. Busacker N. E. (1997). Spatial filtering of multichannel electroencephalographic recordings through principal component analysis by singular value decomposition. J. Clin. Neurophysiol. 14, 73–82. 10.1097/00004691-199701000-00007 9013362

[B30] Lawhern V. David Hairston W. McDowell K. Westerfield M. Robbins K. (2012). Detection and classification of subject-generated artifacts in EEG signals using autoregressive models. J. Neurosci. Methods 208 (2), 181–189. 10.1016/j.jneumeth.2012.05.017 22634706

[B31] Lee S.-H. Lim J. S. Kim J.-K. Yang J. Lee Y. (2014). Classification of normal and epileptic seizure EEG signals using wavelet transform, phase-space reconstruction, and Euclidean distance. Comput. Methods Programs Biomed. 116, 10–25. 10.1016/j.cmpb.2014.04.012 24837641

[B32] Li Y. Ma Z. Lu W. Li Y. (2006). Automatic removal of the eye blink artifact from EEG using an ICA-based template matching approach. Physiol. Meas. 27, 425–436. 10.1088/0967-3334/27/4/008 16537983

[B33] Mahajan R. Morshed B. I. (2015). Unsupervised eye blink artifact denoising of EEG data with modified multiscale sample entropy, kurtosis, and wavelet-ICA. IEEE J. Biomed. Health Inf. 19, 158–165. 10.1109/JBHI.2014.2333010 24968340

[B34] Makinen V. Tiitinen H. May P. (2005). Auditory event-related responses are generated independently of ongoing brain activity. NeuroImage 24, 961–968. 10.1016/j.neuroimage.2004.10.020 15670673

[B35] Mathe M. Padmaja M. Krishna B. T. (2021). Intelligent approach for artifacts removal from EEG signal using heuristic-based convolutional neural network. Biomed. Signal Process. Control 70, 102935. 10.1016/j.bspc.2021.102935

[B36] Min Wanli Luo Gang (2009). Medical applications of EEG wave classification. CHANCE 22 (4), 14–20. 10.1080/09332480.2009.10722978

[B37] Mohammadpoory Z. Nasrolahzadeh M. Haddadnia J. (2017). Epileptic seizure detection in EEGs signals based on the weighted visibility graph entropy. Seizure 50, 202–208. 10.1016/j.seizure.2017.07.001 28732281

[B38] Mumtaz Wajid Rasheed Suleman Irfan Alina (2021). Review of challenges associated with the EEG artifact removal methods. Biomed. Signal Process. Control 68, 102741. 10.1016/j.bspc.2021.102741

[B39] Ng S.-C. Raveendran P. (2009). Enhanced rhythm extraction using blind source separation and wavelet transform. IEEE Trans. Biomed. Eng. 56 (8), 2024–2034. 10.1109/TBME.2009.2021987 19457744

[B40] Onton J. Makeig S. (2006). Information-based modeling of event-related brain dynamics. Prog. Brain Res. 159, 99–120. 10.1016/S0079-6123(06)59007-7 17071226

[B41] Padmavathi K. Sri Ramakrishna K. (2015). Classification of ECG signal during atrial fibrillation using autoregressive modeling. Procedia Comput. Sci. 46, 53–59. 10.1016/j.procs.2015.01.053

[B42] Prado P. F. Mayen M. A. G. Silva G. F. Duarte I. C. S. (2019). Using Matlab's wavelet toolbox to compare electric signals outputted by microbial fuel cells. Sens. Bio-Sensing Res. 24, 100285. 10.1016/j.sbsr.2019.100285

[B43] Rahman F. A. Othman M. F. Shaharuddin N. A. (2015). “A review on the current state of artifact removal methods for electroencephalogram signals,” in 2015 10th Asian Control Conference (ASCC), Kota Kinabalu, Malaysia, 31 May 2015 - 03 June 2015, 1–6. 10.1109/ASCC.2015.7244679

[B44] Richman J. S. Moorman J. R. (2000). Physiological time-series analysis using approximate entropy and sample entropy. Am. J. Physiol. Heart Circ. Physiol. 278, H2039–H2049. 10.1152/ajpheart.2000.278.6.H2039 10843903

[B45] Rissanen J. (1999). Stochastic complexity in statistical inquiry. Washington, DC: Government Printing Office. World Scientific; 1989. U.S. Department of Defense Office of the Secretary of Defense. Code of federal regulations, protection of human subjects. 32 CFR 219.

[B46] Rodr´ıguez-Berm´udez G. Garcia-Laencina P. J. (2015). Analysis of eeg signals using nonlinear dynamics and chaos: A review. Appl. Math. Inf. Sci. 9 (5), 2309.

[B47] Romero S. Ma?nanas M. Barbanoj M. (2008). A comparative study of automatic techniques for ocular artifact reduction in spontaneous EEG signals based on clinical target variables: A simulation case. Comput. Biol. Med. 38 (3), 348–360. 10.1016/j.compbiomed.2007.12.001 18222418

[B48] Romo Vázquez R. Vélez-Pérez H. Ranta R. Louis Dorr V. Maquin D. Maillard L. (2012). Blind source separation, wavelet denoising and discriminant analysis for EEG artefacts and noise cancelling. Biomed. Signal Process. Control 7 (4), 389–400. 10.1016/j.bspc.2011.06.005

[B49] Sadeghi Bajestani G. Hashemi Golpayegani M. R. Sheikhani A. Ashrafzadeh F. (2017). Poincaré section analysis of the electroencephalogram in autism spectrum disorder using complement plots. Kybernetes 46 (2), 364–382. 10.1108/K-12-2015-0306

[B50] Sai C. Y. Mokhtar N. Arof H. Cumming P. Iwahashi M. (2018). Automated classification and removal of EEG artifacts with SVM and wavelet-ICA. IEEE J. Biomed. Health Inf. 22 (3), 664–670. 10.1109/JBHI.2017.2723420 28692997

[B51] Sanei S. Chambers J. A. (2007). EEG signal processing. NewYork: Wiley.

[B52] Sayed K. Kamel M. Alhaddad M. Malibary H. M. Kadah Y. M. (2017). Characterization of phase space trajectories for Brain-Computer Interface. Biomed. Signal Process. Control 38, 55–66. 10.1016/j.bspc.2017.05.007

[B53] Senthilkumar P. (2008). Removal of ocular artifacts in the EEG through wavelet transform without using an EOG reference channel. Int. J.Open Probl. Compt. Math. 1 (3), 188–200.

[B54] Seppänen J. Turunen J. Koivisto M. Haarla L. (2015). Measurement based analysis of electromechanical modes with second order blind identification. Electr. Power Syst. Res. 121, 67–76. 10.1016/j.epsr.2014.11.023

[B55] Sharif B. Homayoun Jafari A. (2017). Prediction of epileptic seizures from EEG using analysis of ictal rules on Poincaré plane. Comput. Methods Programs Biomed. 145, 11–22. 10.1016/j.cmpb.2017.04.001 28552116

[B56] Sharma R. Pachori R. B. (2015). Classification of epileptic seizures in EEG signals based on phase space representation of intrinsic mode functions. Expert Syst. Appl. 42 (3), 1106–1117. 10.1016/j.eswa.2014.08.030

[B57] Sharma R. Pachori R. B. Acharya U. R. (2015). Application of entropy measures on intrinsic mode functions for the automated identification of focal electroencephalogram signals. Entropy 17, 669–691. 10.3390/e17020669

[B58] Shoker L. Sanei S. Chambers J. (2005). Artifact removal from electroencephalograms using a hybrid BSS-SVM algorithm. IEEE Signal Process. Lett. 12, 721–724. 10.1109/LSP.2005.855539

[B59] Sun W. Su Y. Wu X. Wu X. (2020). A novel end-to-end 1D-ResCNN model to remove artifact from EEG signals. Neurocomputing 404, 108–121. 10.1016/j.neucom.2020.04.029

[B60] Sweeney K. T. McLoone S. F. Ward T. E. (2013). The use of ensemble empirical mode decomposition with canonical correlation analysis as a novel artifact removal technique. IEEE Trans. Biomed. Eng. 60 (1), 97–105. 10.1109/TBME.2012.2225427 23086501

[B61] Takens F. (1981). “Detecting strange attractors in turbulence,” in Dynamical systems and turbulence, Warwick 1980. Editors Rand D. Young L.-S. (Springer), 366–381. Lecture notes in mathematics.

[B62] Tang A. C. yyimuner B. A. Malaszenko N. A. Phung D. B. Reeh B. C. (2002a). Independent components of magnetoencephalography: Localization. Neural Comput. 14, 1827–1858. 10.1162/089976602760128036 12180404

[B63] Tang A. C. Pearlmuger B. A. Malaszenko N. A. Phung D. B. (2002b). Independent components of magnetoencephalography: Single-trial response onset times. Neuroimage 17, 1773–1789. 10.1006/nimg.2002.1320 12498751

[B64] Taskinen S. Miettinen J. Nordhausen K. (2016). A more efficient second order blind identification method for separation of uncorrelated stationary time series. Statistics Probab. Lett. 116, 21–26. 10.1016/j.spl.2016.04.007

[B65] Vigário R. Oja E. (2000). Independence: A new criterion for the analysis of the electromagnetic fields in the global brain? Neural Netw. 13 (8–9), 891–907. 10.1016/S0893-6080(00)00073-3 11156200

[B66] Vorobyov S. Cichocki A. (2002). Blind noise reduction for multisensory signals using ICA and subspace filtering, with application to EEG analysis. Biol. Cybern. 86, 293–303. 10.1007/s00422-001-0298-6 11956810

[B67] Wam Y. Sutherland M. T. Lori L. (2004). “Single-trial classification of ERPs using second order blind identification (SOBI),” in Proceedings of the Third International Conference on Machine Leaming and Cybernetics, Shanghai, 26-23 August 2004.

[B68] Webb L. Kauppila M. Roberts J. A. Vanhatalo S. Stevenson N. J. (2021). Automated detection of artefacts in neonatal EEG with residual neural networks. Comput. Methods Programs Biomed. 208, 106194. 10.1016/j.cmpb.2021.106194 34118491

[B69] Yang Banghua Duan Kaiwen Fan Chengcheng Hu Chenxiao Wang Jinlong (2018). Automatic ocular artifacts removal in EEG using deep learning. Biomed. Signal Process. Control 43, 148–158. 10.1016/j.bspc.2018.02.021

[B70] Yeung N. Bogacz R. Holroyd C. B. Nieuwenhuis S. Cohen J. D. (2007). Theta phase resetting and the error-related negativity. Psychophysiology 44, 39–49. 10.1111/j.1469-8986.2006.00482.x 17241139

[B71] Zangeneh Soroush M. Maghooli K. Kamaledin Setarehdan S. Nasrabadi A. M. (2019a). Emotion recognition through EEG phase space dynamics and Dempster-Shafer theory. Med. Hypotheses 127, 34–45. 10.1016/j.mehy.2019.03.025 31088645

[B72] Zangeneh Soroush M. Maghooli K. Setarehdan S. K. Nasrabadi A. M. (2017). A review on EEG signals based emotion recognition. Int. Clin. Neurosci. J. 4 (4), 118–129. https://journals.sbmu.ac.ir/neuroscience/article/view/18477. 10.15171/icnj.2017.01

[B73] Zangeneh Soroush M. Maghooli K. Setarehdan S. K. Nasrabadi A. M. (2020). Emotion recognition using EEG phase space dy-namics and Poincare intersections. Biomed. Signal Process. Control 59, 101918. 10.1016/j.bspc.2020.101918

[B74] Zangeneh Soroush M. Maghooli K. Zanganeh Soroush P. Tahvilian P. Bagherzadeh S. (2018a). EEG-based emotion recognition through nonlinear analysis. Int. J. Sci. Eng. Invest. 7, 62–69.

[B75] Zangeneh Soroush M. (2021). Nonlinear electroencephalogram (EEG) analysis in sleep medicine. J. Sleep. Sci. 5 (3), 122–123.

[B76] Zangeneh Soroush M. Maghooli K. Setarehdan S. K. Nasrabadi A. M. (2018b). A novel approach to emotion recognition using local subset feature selection and modified Dempster-Shafer theory. Behav. Brain Funct. 14, 17. 10.1186/s12993-018-0149-4 30382882 PMC6208176

[B77] Zangeneh Soroush M. Maghooli K. Kamaledin Setarehdan S. Nasrabadi A. M. (2018c). A novel method of EEG-based emotion recognition using nonlinear features variability and dempster-shafer theory, biomedicalengineering: Applications. Biomed. Eng. Appl. Basis Commun. 30 (2), 1850026. 10.4015/S1016237218500266

[B78] Zangeneh Soroush M. Maghooli K. Setarehdan S. K. Nasrabadi A. M. (2018d). Emotion classification through nonlinear EEG analysis using machine learning methods. Int. Clin. Neurosci. J. 205 (4), 135–149. https://journals.sbmu.ac.ir/neuroscience/article/view/22921. 10.15171/icnj.2018.26

[B79] Zangeneh Soroush M. Maghooli K. Setarehdan S. K. Nasrabadi A. M. (2019b). A novel EEG-based approach to classify emotions through phase space dynamics. Signal Image Video process. 13, 1149–1156. 10.1007/s11760-019-01455-y

[B80] Zhang H. Zhao M. Chen W. Mantini D. Li Z. Liu Q. (2021). EEGdenoiseNet: A benchmark dataset for deep learning solutions of EEG denoising. J. Neural Eng. 18 (5), 056057. 10.1088/1741-2552/ac2bf8 34596046

[B81] Zikov T. Bibian S. Dumont G. Huzmezan M. Ries C. (2002). “A wavelet based de-noising technique for ocular artifact correction of the electroencephalogram,” in Proceedings of the Second Joint 24th Annual Conference and the Annual Fall Meeting of the Biomedical Engineering Society] [Engineering in Medicine and Biology, 23-26 October 2002, Houston, TX, USA, 98–105.

[B82] Zou Y. Donner R. V. Marwan N. Donges J. F. Kurths J. (2019). Complex network approaches to nonlinear time series analysis. Phys. Rep. 787, 1–97. 10.1016/j.physrep.2018.10.005

