# Supplementary figures and images for: EEG artifact removal using sub-space decomposition, nonlinear dynamics, stationary wavelet transform and machine learning algorithms
Source: Front Physiol. 2022 Aug 24;13:910368. doi: 10.3389/fphys.2022.910368 (PMC9449652; doi:10.3389/fphys.2022.910368)

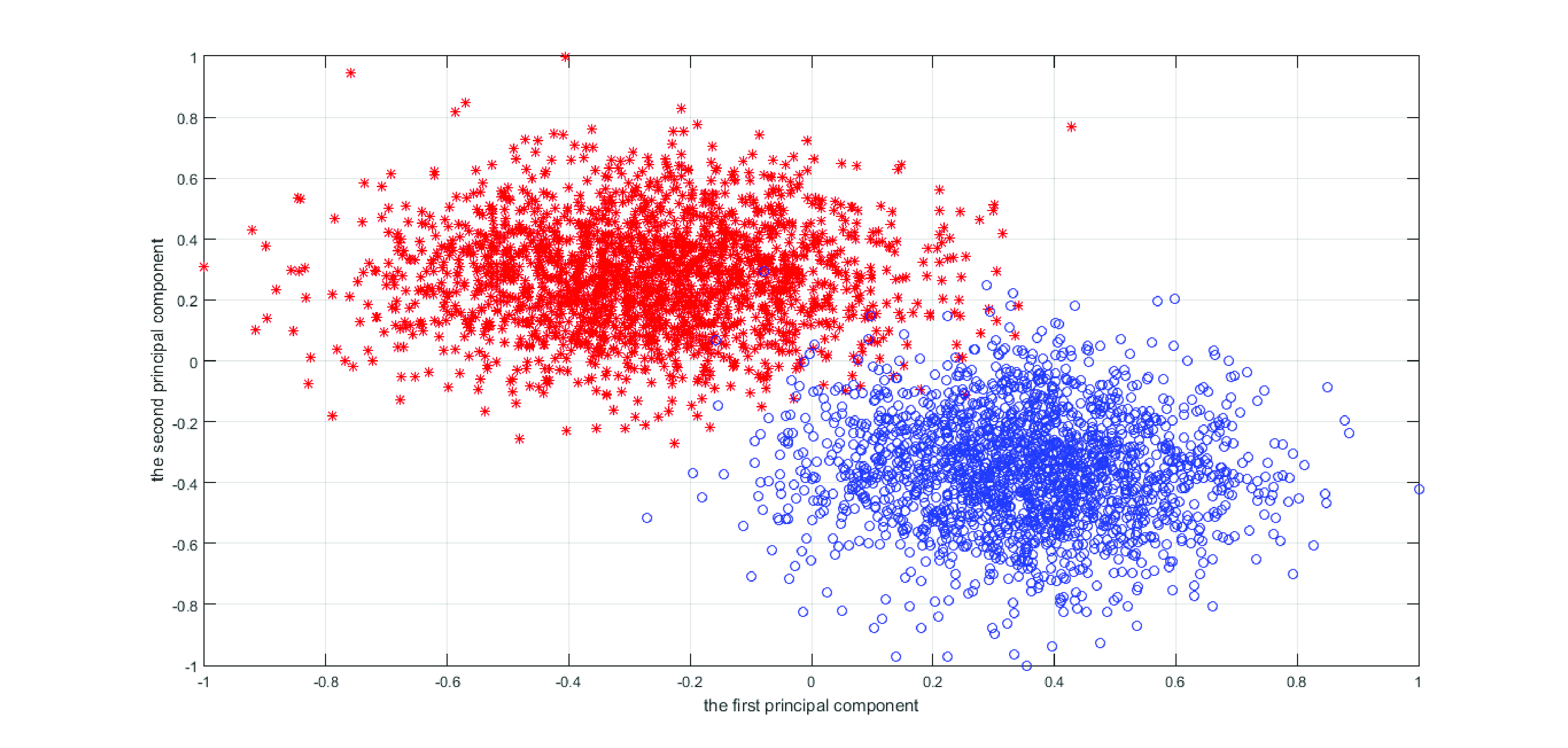

Supplement: Supplementary file 1 [file Image2.jpg]

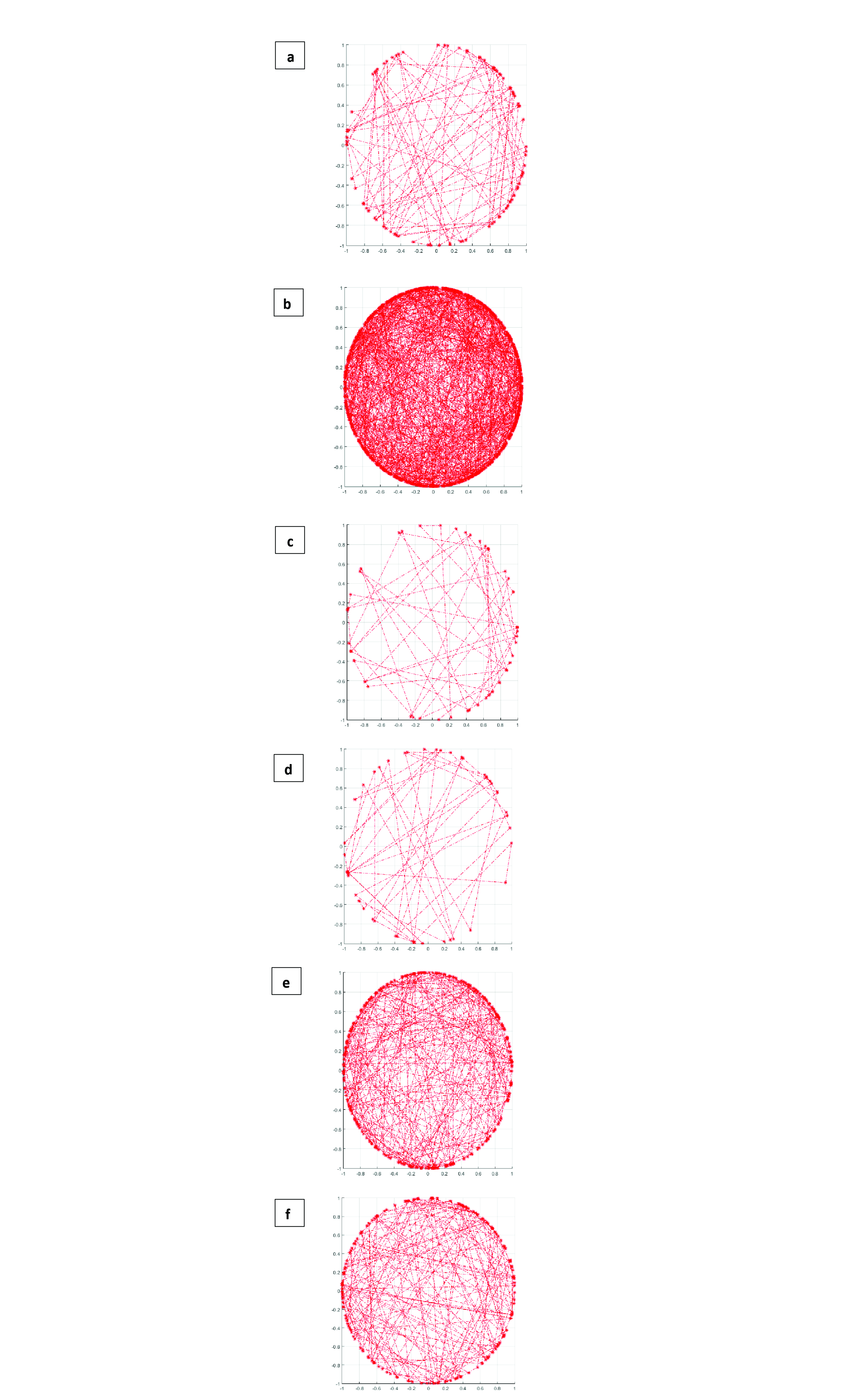

Supplement: Supplementary file 3 [file Image1.jpg]
