# Supplementary material for: EEG artifact removal using sub-space decomposition, nonlinear dynamics, stationary wavelet transform and machine learning algorithms
Source: Front Physiol. 2022 Aug 24;13:910368. doi: 10.3389/fphys.2022.910368 (PMC9449652; doi:10.3389/fphys.2022.910368)
Supplement: Supplementary file 2 [file Table1.pdf]

Table 8. Average processing time at signal length 10 s, 30 s and 60 s over all components

Signal length	10 s	30 s	60 s
Processing time	0.08 ± 0.02	0.17 ± 0.03	0.23 ± 0.05
